# Strong gradients, cool performance: A 64‐channel array coil with concurrent field monitoring and thermal control for ex vivo diffusion‐weighted brain imaging using the 3T connectome 2.0 MRI scanner

**DOI:** 10.1002/mrm.30599

**Published:** 2025-06-08

**Authors:** Alina Müller, Mirsad Mahmutovic, Mona Alem, Gabriel Ramos‐Llordén, Dongsuk Sung, Manisha Shrestha, Sam‐Luca J.D. Hansen, Chaimaa Chemlali, Anpreet Ghotra, Jason Stockmann, Choukri Mekkaoui, Lawrence L. Wald, Anastasia Yendiki, Susie Y. Huang, Boris Keil

**Affiliations:** ^1^ Institute of Medical Physics and Radiation Protection TH Mittelhessen University of Applied Sciences Giessen Hesse Germany; ^2^ Athinoula A. Martinos Center for Biomedical Imaging, Department of Radiology Massachusetts General Hospital, Harvard Medical School Boston Massachusetts USA; ^3^ Harvard‐MIT Division of Health Sciences and Technology Massachusetts Institute of Technology Cambridge Massachusetts USA; ^4^ Department of Diagnostic and Interventional Radiology University Hospital Marburg, Philipps University of Marburg Hesse Germany; ^5^ LOEWE Research Cluster for Advanced Medical Physics in Imaging and Therapy (ADMIT) TH Mittelhessen University of Applied Sciences Giessen Hesse Germany

**Keywords:** diffusion MRI, field monitoring, human connectome, phased array coil, radiofrequency coil

## Abstract

**Purpose:**

High‐resolution ex vivo diffusion‐weighted imaging (dMRI) with high b‐values presents significant challenges, including low signal‐to‐noise ratio (SNR), magnetic field perturbations, and temperature‐related measurement shifts. This work introduces a hardware‐based solution to address these limitations in human ex vivo brain imaging.

**Methods:**

A customized anatomically conformal 64‐channel receive array coil with a dedicated Tx birdcage coil was developed for 3T diffusion‐weighted imaging of whole human ex vivo brain specimens. Field monitoring capabilities were integrated to correct spatiotemporal field perturbations caused by gradient‐induced eddy currents. Temperature stability throughout extended acquisition periods was achieved through an integrated stabilization system. Coil performance was validated through comprehensive measurement of SNR, g‐factor maps, field camera free induction decays (FIDs), temperature, mean diffusivity, and fractional anisotropy across multiple diffusion‐weighted scans.

**Results:**

The system demonstrated 73% higher SNR compared with a 72‐channel in vivo head coil. Integration of the field camera maintained its FID quality without SNR penalties or significant receive coil coupling effects. Temperature stabilization improved the reliability of quantitative diffusion‐weighted measurements by eliminating measurement drift during a 13‐hour acquisition, where mean diffusivity and mean kurtosis would have increased by 22% and decreased by 19%, respectively.

**Conclusion:**

We describe an integrated hardware approach for addressing higher order field perturbations, thermal instability, and SNR challenges in human ex vivo whole brain dMRI under high‐diffusion sensitizing gradient conditions. This approach combines an anatomically optimized multichannel receive array, concurrent field monitoring, and active temperature stabilization. Enhanced image quality and improved reliability of quantitative MR imaging were demonstrated with this comprehensive hardware solution.

## 1 INTRODUCTION

Understanding the intricate neural networks of the human brain is fundamental to unraveling the complexities of human behavior and neurological diseases. Diffusion‐weighted magnetic resonance imaging (dMRI) has emerged as a pivotal tool in this process. It exploits the anisotropic diffusion of water molecules in brain tissue to reveal the orientation of neural fiber pathways at the voxel level, primarily within white matter tracts. However, achieving submillimeter spatial resolution necessary for detailed connectome mapping, is fraught with challenges, primarily due to limitations in signal‐to‐noise ratio (SNR) and the need for high b‐value diffusion data acquisitions.

Several strategies can address these challenges, including the utilization of high‐performance MRI gradient systems,^1^, ^2^, ^3^, ^4^, ^5^ long‐duration ex vivo brain imaging,^4^, ^6^, ^7^, ^8^, ^9^, ^10^, ^11^, ^12^, ^13^ dedicated target‐specific receive coils,^6^, ^14^, ^15^, ^16^, ^17^ and high field strength MRI.^3^, ^4^, ^18^, ^19^, ^20^ In this study, we focus on three key aspects: First, we used the Connectome 2.0 scanner with its high gradient strength $G_{\max}$ of 500 mT/m and slew rates ($SR_{\max}$) of 600 T/m/s, enhancing high b‐value diffusion encoding.^21^, ^22^ Second, we utilized post‐mortem ex vivo diffusion MRI. Because ex vivo MRI can accommodate extended scan durations, it is capable of attaining significantly enhanced spatial and angular resolution, thereby allowing the delineation and characterization of intricate fiber pathways' anatomy and microstructure at the submillimeter scale.^23^, ^24^ This surpasses the resolution attainable through in vivo imaging techniques and narrows the gap between macroscopic imaging and microscopic histology. Third, we introduced a newly developed human brain‐shaped ex vivo brain radiofrequency (RF) coil tailored specifically for the application of high spatial resolution and high b‐value dMRI with the Connectome 2.0 scanner.

Despite these advancements, the challenges of high‐resolution *ex vivo* diffusion extend beyond SNR and image resolution. Particularly when using strong diffusion‐sensitizing gradients, eddy currents and concomitant Maxwell terms occur, resulting in spatiotemporally and dynamically varying nonlinear magnetic fields dynamics, causing substantial image artifacts such as ghosting, blurring, and distortion.^8^, ^25^, ^26^ Conventional methods for artifact reduction are often ineffective under these conditions.^8^, ^27^, ^28^ To mitigate these unwanted field perturbations, concurrent field monitoring systems have proven valuable.^26^, ^29^, ^30^, ^31^ This involves using a field camera setup^32^ to capture spatial and temporal magnetic field dynamics of the imaging target volume, utilizing a spherical harmonic field model.^33^, ^34^, ^35^ This technique facilitates the correction of encoding trajectories during image reconstruction, thus minimizing artifacts and enhancing image quality. It has already been successfully applied for dMRI with b‐values up to 10,000 s/mm^2^ and gradient strengths up to 268 mT/m in ex vivo imaging.^36^ An external field camera was used to measure field dynamics in a separate prescan acquisition. Integrated approaches incorporating field probes have also been explored in conjunction with in vivo head coils.^26^, ^29^, ^30^, ^31^, ^37^, ^38^, ^39^, ^40^ Another critical challenge in ex vivo dMRI is the management of sample temperature during long scans. Ex vivo brain specimens lack natural thermoregulatory mechanisms, leading to gradual heating due to the energy absorbed from MRI transmit pulses and heat dissipation from the surrounding coil electronics. This heating can significantly alter diffusion measurements, since fractional anisotropy (FA) and apparent diffusion coefficient (ADC) are temperature dependent.^41^ Thus, monitoring and maintaining a stable sample temperature throughout the long‐duration scanning process is essential for reliable diffusion measurements.^42^ The primary objective of this work was to develop and orchestrate an instrumentation setup that was capable of overcoming the challenges associated with strong diffusion‐sensitizing gradients and prolonged ex vivo scans of entire human brain specimens. We designed, constructed, and evaluated a human brain‐shaped receive array coil comprising 64 detector elements, complemented by a dedicated circularly polarized transmit birdcage coil, for ex vivo dMRI applications with the 3T Connectome 2.0 scanner. Additionally, the coil system includes an integrated concurrent 16‐channel field monitoring system to account for nonlinear dynamic magnetic field changes caused by the high‐performance gradient coil. The incorporation of temperature probes and a forced airflow mechanism ensures that the brain specimen's temperature remains stable during long MRI scans. This approach aims to provide an advanced environment for accurate and reliable high‐resolution, high b‐value dMRI ex vivo data acquisition over extended durations, pushing the boundaries of human Connectome mapping.

## 2 METHODS

### 2.1 Coil design

#### 2.1.1 Coil former and receive array

**FIGURE 1:**
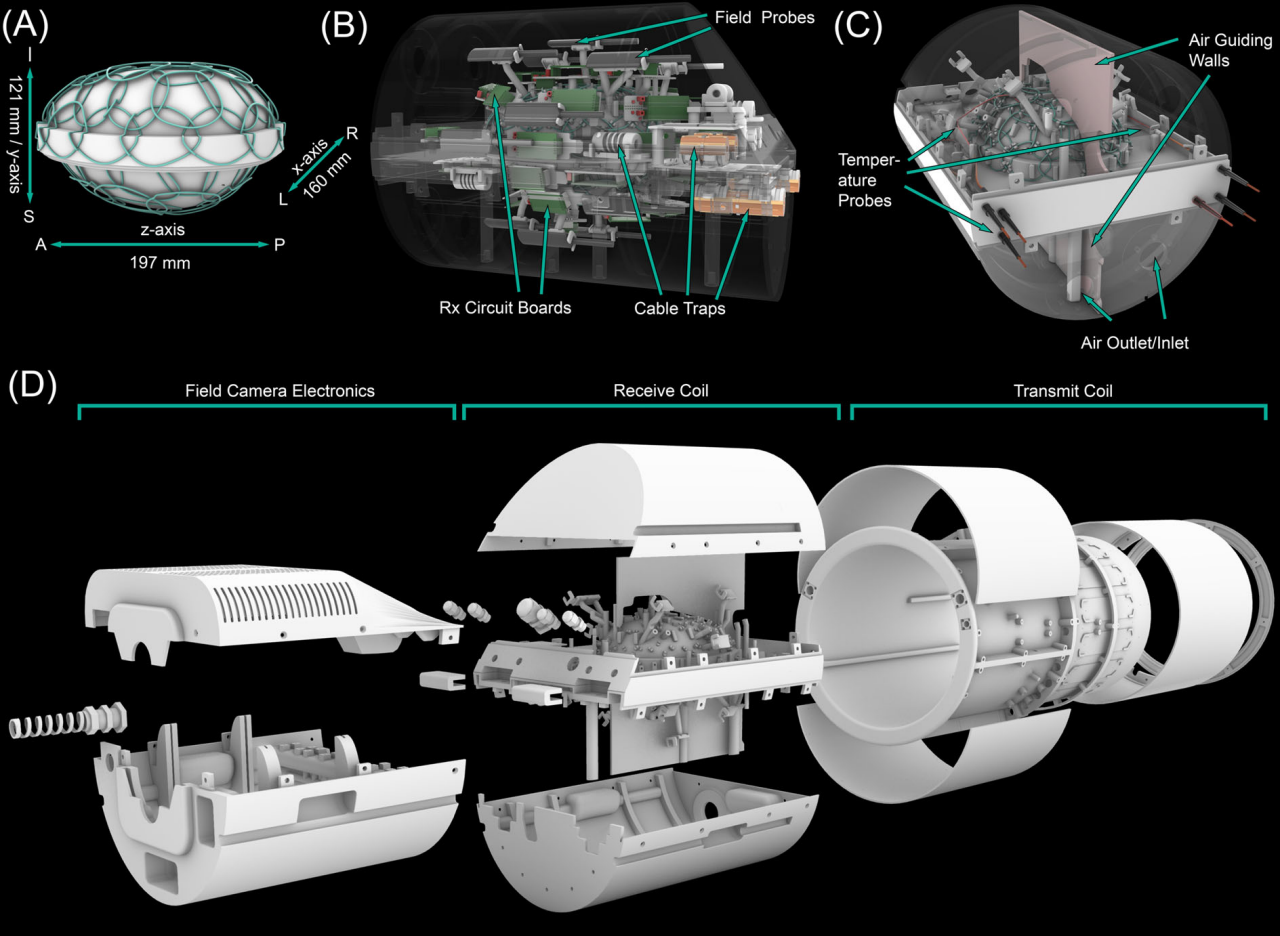
CAD model of the 64‐channel ex vivo brain coil (A) Coil former with 64 loops and coil former dimensions (anatomical axes of the brain) from left (L) to right (R), anterior (A) to posterior (P), and superior (S) to inferior (I). Note that the anatomical axes do not correspond to the normal positioning of the brain in the scanner, as the brain container is rotated 90 degrees around the x‐axis compared with in vivo positioning. The top and bottom coil housing parts mechanically overlap to allow adjacent coil elements on the two halves to be geometrically decoupled. (B) Coil design with loops and related Rx components, field probes and various mountings. (C) Design of the temperature stabilization system. (D) Exploded view of mechanical parts of the coil, including strain reliefs and hinges as factory made components, while all other parts shown were 3D printed.

**FIGURE 2:**
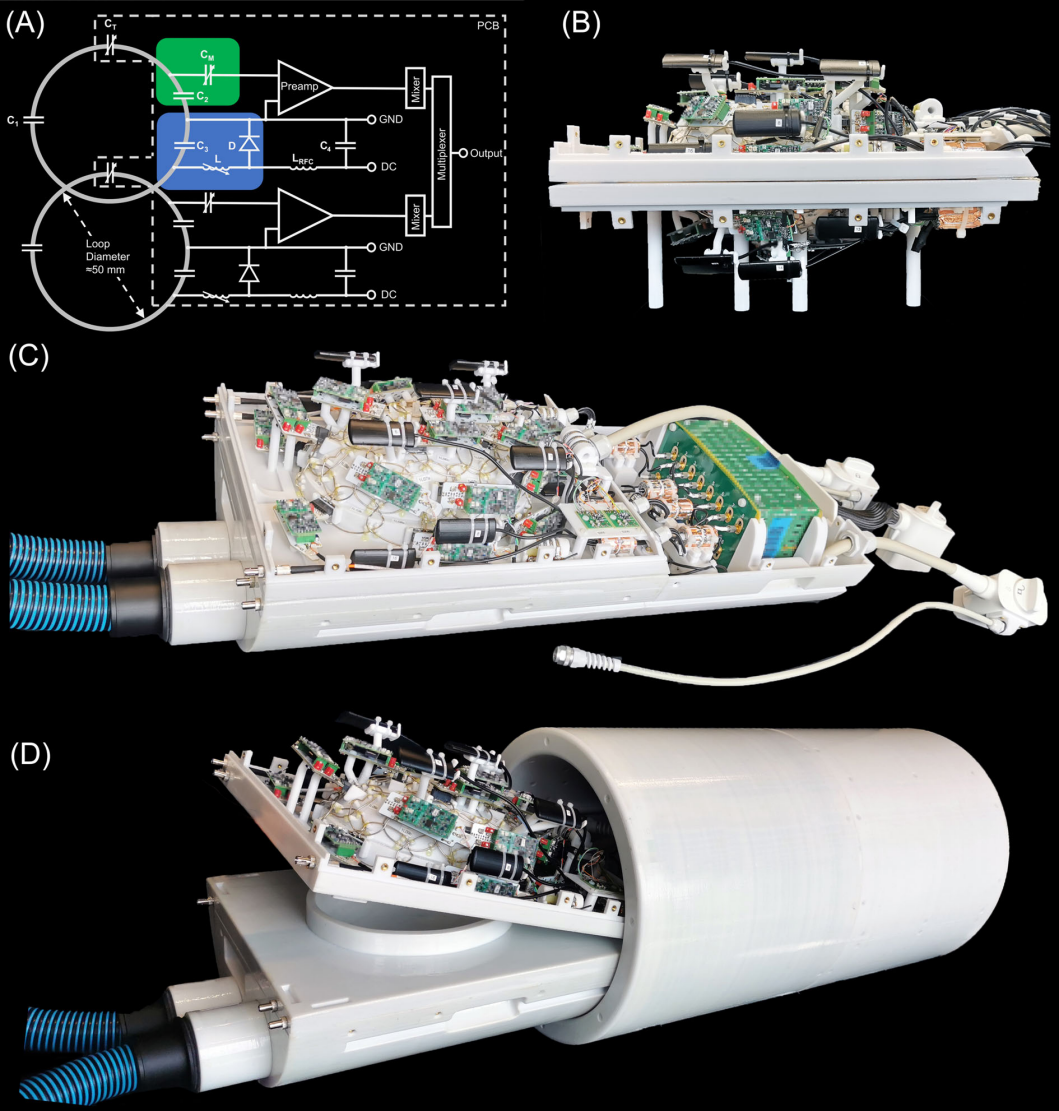
(A) Circuit schematic of two coil elements with matching network (green) and active detuning circuit (blue). Typical capacitor values are: $C_{1}=24$ pF, $C_{2}=62$ pF, $C_{3}=56$ pF, $C_{T}=13$ pF, $C_{M}=19$ pF. (B) Top and bottom part of the constructed 64‐channel ex vivo brain coil with integrated field probes. (C) Complete constructed Rx coil with field camera, temperature probes, bottom air hoses and field camera in‐bore first stage electronics box. (D) Rx coil within the Tx birdcage coil. A tilting mechanism with a hinge is integrated for top and bottom coil part to facilitate both insertion of the brain and cable connection between the top part and the field camera in‐bore electronics box, which is located in the bottom part.

The mechanical design of the coil former extends and refines the concept of our previous work,^6^ incorporating improvements in shape and size, increasing the coil element count to 64 receive (Rx) channels (Figure 1A), and adding functionalities for dynamic magnetic field and temperature monitoring (Figures 1B,C and 2B–D). The coil is designed to accommodate ex vivo brain sizes up to the 97th percentile of the male population, with an overall volume of 2164 cm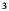. The coil former's shape accounts for the typical oblate deformation that occurs when the brain is not supported by the skull. Consequently, the coil former conforms to the ex vivo brain's shape, accommodating both cerebral hemispheres and the cerebellum, resulting in an oblate spheroid container (197 mm × 160 mm × 121 mm) that encloses the entire brain and ensures placement of the sample in the scanner's isocenter. The housing is divided into two separate mechanically overlapping sections: an upper part with 38 coil elements and a lower section comprising 26 coil elements. These sections are connected by hinges, allowing for convenient positioning of the ex vivo brain sample within the coil former. This midsection housing split incorporates an overlapping mechanical rim‐like structure to ensure proper closure of the two segments. The overlapping portion was also precisely designed to meet the specifications for critical overlap of the receive coil elements located at the edges of the two sections. The position and shape of each loop element were allocated with incorporated positioners onto the former, facilitating the accurate construction of the detector array. All mechanical coil parts (Figure 1D) were printed in polycarbonate plastic using rapid prototyping 3D printing technology (Fortus‐Series, Stratasys, Eden Prairie, MN, USA).

#### 2.1.2 Rx circuitry

Both the receive array and the transmit (Tx) coil were carefully designed to optimize RF performance while minimizing eddy current induction, by a strategic selection of materials and geometric configurations. The circular loop elements were constructed using 1.5‐mm silver‐plated copper wire. Wire was utilized instead of flat conductors to reduce eddy current losses.^43^ The loops were critically overlapped to geometrically decouple adjacent detector elements (Figure 1A). The loop diameters ranged from 48 mm to 52 mm. Each loop element consisted of semicircular wire halves joined by a tuning capacitor and an interconnecting pin connector. This connector linked the loop elements to a printed circuit board (PCB), which housed the RF electronic components that were necessary for completing the resonant structure of the elements, including matching, active and passive detuning networks, and preamplification. In terms of eddy current reduction, the PCBs were designed without large copper structures. Figure 2A provides the circuit schematic. Further technical information of the circuit components is provided in Table 1. In brief, $C_{1}$, $C_{2}$, $C_{3}$, and $C_{T}$ are the primary tuning capacitors. $C_{T}$ was used to accurately fine‐tune the resonant circuit to the Larmor frequency of 123.25 MHz. Capacitor $C_{M}$, along with the variable capacitor $C_{2}$, facilitated matching to 50 Ω,^44^ while also establishing preamplifier decoupling to impart a serial high impedance into the loop, thereby suppressing current flow at the Larmor frequency.^45^ This method effectively decoupled the non‐neighboring loop elements throughout the coil array. Capacitor $C_{3}$, in conjunction with the variable inductance L and the PIN diode D, formed the active detuning circuit when the PIN diode was forward biased during RF transmission.^46^ Pairs of loop elements were grouped and connected to a twin preamplifier (Siemens Healthineers AG, Forchheim, Germany), which multiplexed the two coils' RF signals onto a single coaxial output cable.^47^ These coaxial cables were bundled and passed through solenoid cable traps to suppress RF common mode currents induced from the transmit system. Two standard coil system plugs connect the ex vivo coil to the MRI scanner's patient bed.

**TABLE 1:** Components of the Rx and Tx circuits and cable traps.

	Component	Model number	Manufacturer	Value
Rx	$C_{T}$, $C_{M}$	JR200	Voltronics Corp., Danville, NJ, USA	4.5–20 pF
	$C_{1}$, $C_{2}$, $C_{3}$	Series 11	Voltronics Corp., Danville, NJ, USA	see Figure S2
	$C_{4}$	VJ1206A222JCXAT	Vishay Intertechnology, Inc.	2.2 nF
	L	165‐02A06L	Coilcraft Inc., Cary, IL, USA	25‐32 nH
	D	MA4P4002B‐402	Macom, Lowell, MA, USA	—
	$L_{\text{RFC}}$	1812CS‐333XJLCt	Coilcraft Inc., Cary, IL, USA	2.7 μH
Tx	$C_{\text{ER}}$	Series 25	Voltronics Corp., Danville, NJ, USA	43 pF
	$C_{\text{sym}}$	Series 25	Voltronics Corp., Danville, NJ, USA	1–2 pF
	$C_{R}$	Series 11	Voltronics Corp., Danville, NJ, USA	150 pF
	$D_{\text{Tx}}$	A4P7470F‐1072T	Macom, Lowell, MA)	—
	$L_{\text{RFC}}$	1812CS‐333XJLCt	Coilcraft Inc., Cary, IL, USA	2.7
	$C_{\text{TTx}}$	Series 25	Voltronics Corp., Danville, NJ, USA	1–2 pF
	$C_{\text{MTx}}$	Series 25	Voltronics Corp., Danville, NJ, USA	62 pF
	$C_{S}$	Series 11	Voltronics Corp., Danville, NJ, USA	1 nF
traps	$C_{\text{st}}$	Series 25	Voltronics Corp., Danville, NJ, USA	33–62 pF
	$C_{\text{ft}}$	Series 11	Voltronics Corp., Danville, NJ, USA	15–39 pF

*Note*: For the cable traps $C_{\text{st}}$ denotes the capacitance of the solenoid trap and $C_{\text{ft}}$ represents the capacitance of the floating shield trap.

#### 2.1.3 Field camera

Given the various sources of magnetic field perturbations when applying strong diffusion‐sensitizing gradient pulses, higher order field dynamic monitoring becomes crucial for image quality. Effective monitoring of magnetic field dynamics up to the 3rd order across a volume of interest can be achieved by strategically placing 16 field probes around the volume and utilizing spherical harmonics expansion.^25^, ^48^ We integrated a commercially available magnetic field camera (Skope MR Technologies Inc., Zurich, Switzerland) into the ex vivo coil system, positioning the 16 ^19^F transmit/receive NMR field probes around the coil former. The placement conditions focused on (1) minimizing the noise standard deviation of the field probes' phase‐error coefficients for accurate spherical harmonics expansion, (2) maintaining sufficient distance from other electrical coil components such as preamplifiers and cables to reduce mutual interactions, (3) avoiding any nearby materials that could potentially modulate the susceptibility in the proximity of the NMR‐sensitive fluorine droplet, and (4) relatively low distance from the isocenter to reduce signal reduction due to high dephasing through strong gradients. This optimization process was conducted within a space‐constrained environment of a 31‐cm inner diameter of the transmit coil.

Building on an initial field probe distribution outlined by Barmet et al.^25^, ^39^ for an optimal head coil configuration, we iteratively rearranged the field probes and coil components using a 3D computer‐aided design (CAD) environment before the actual construction process. Each potential geometric probe configuration was meticulously evaluated by computing the noise standard deviation and the maximum phase errors^39^ derived from an obtained probing matrix P,^33^ and the spherical harmonics up to the 3rd order^35^ for capturing the magnetic field within a 20‐cm diameter sphere. We evaluated various probe arrangements, including more omni‐directional distributions around the brain specimen, iteratively progressing toward an optimized solution. The final optimized probe configuration comprised four z‐stacked rings with 6‐5‐4‐1 distributed probes per ring, averaging a distance of 14 cm from the scanner isocenter.

With the optimized probe configuration established, we designed the associated mountings and cable guides for the camera probes to complement the overall mechanical design of the ex vivo coil array. To suppress common mode currents from the Tx system, floating shield ^1^H cable traps^49^ were incorporated into the probes' cable arrangement. These cable traps feature a slotted design to reduce eddy currents. The first stage electronics of the field camera were also integrated into the ex vivo coil assembly (Figure 2C), with special attention to housing ventilation to prevent heat build‐up from the active electronic parts. A standard coil system plug was used to route the camera's cable bundle alongside the regular RF coil cables within the MRI patient table, connecting the field camera's front end to its dedicated NMR spectrometer located outside the scanner's RF‐shielded cabin.

#### 2.1.4 Temperature stabilization

Stabilizing the ex vivo sample's temperature during prolonged diffusion scans requires the installation of on‐coil temperature monitoring probes and a forced‐air system to consistently maintain the coil's ambient temperature inside the housing structure. We installed four 1.5mm‐thick fluorescence‐based fiber‐optic temperature probes (PRB‐100‐STM‐MRI, OSENSA Innovations Corp. Burnaby, BC, Canada, stability: 0.3°C/24h, peak‐to‐peak noise: 0.02°C) inside the coil housing to measure the ambient temperature outside the ex vivo sample compartment near the air inlet and the air outlet in both coil parts. Additionally, two temperature probes were placed directly inside the ex vivo sample compartment to monitor the peripheral temperature of the ex vivo brain samples. The measured temperature signals were routed via fiber optic cables through the MRI cabin's waveguide to an optoelectronic signal‐processing station (FTX‐300‐LUX, OSENSA Innovations Corp. Burnaby, BC, Canada) in the MRI control room.

A duct air blower (19135K66, McMaster‐Carr, Elmhurst, IL), also located in the control room, was connected to an air hose system routed through the waveguide into the scanner room and connected to the upper and lower ex vivo coil housing segments with a bayonet fitting (Figure S1). The inner coil housing was equipped with air‐guiding internal walls, ensuring constant airflow from the input opening to the output opening on the opposite housing side.

#### 2.1.5 Transmit birdcage coil

The Connectome 2.0 MRI scanner lacks a built‐in transmit coil system, necessitating the use of dedicated local Tx coils for any MRI scan. Consequently, a Tx birdcage coil was constructed with a design that accommodates geometric integration of the ex vivo 64‐channel Rx array. The Tx coil, a bandpass birdcage with quadrature drive, features 16 rungs, two end rings, and a central ring structure for distributing the bias currents to each PIN diode. The coil has a diameter of 318 mm, a length of 236 mm, and a shield with a diameter of 363 mm and a length of 280 mm (see more details and circuit in Figures S2 and S3). Eddy current reduction was achieved by segmenting large copper surfaces on the rungs and birdcage shield (Figure S4). Additionally, the copper thickness of the slotted RF shield was set to 9 μm, providing sufficient skin depth for RF mirror currents on the shield while offering poor‐conditioned conductivity depth for low‐frequency eddy currents. To maintain RF mirror current continuity on the shield, we placed 1 nF ceramic capacitors exclusively along the RF mirror current path. The optimal segmentation pattern was determined through simulation using a hybrid Method‐of‐Moments and Finite‐Element‐Method solver (FEKO v. 2021.2, Altair Engineering, Troy, Michigan, USA). For the simulation, we incorporated the designs of the x‐, y‐, and z‐gradient coils with a 30‐kHz sinusoidal waveform, adjusting the drive set amplitude to achieve a gradient strength of 500 mT/m per axis.

Mechanically, the Rx coil structure must be removable from the Tx birdcage coil to place the ex vivo brain sample in the coil array. We integrated rails inside the birdcage coil and created a matching groove pattern on the Rx coil structure, establishing a sliding mechanism that facilitates brain sample placement with relative ease.

### 2.2 Bench measurements

The bench measurements of the ex vivo coil array included the analysis of tuning, matching, active detuning, and preamplifier decoupling. A custom‐made coil plug simulator was used, providing 3 V for the preamplifiers and the capability to forward‐bias the PIN diode of the coil element's active detuning circuit with 100 mA. To facilitate rapid switching between the required termination states of each loop coil, preamplifier dummy boards were used.^50^ These boards provided 50 Ω termination, the complex input impedance of the preamplifier, and a pass‐through connection for direct $S_{11}$ measurements.

During the array's tune‐up process, all ^19^F field probes were present to account for potential influences on the coil elements. Tuning and matching for each coil element were adjusted under $S_{11}$ control using a custom‐built brain‐shaped phantom as a load (closely following the phantom recipe from the FBIRN study^51^: 1.7 L H_2_O, 0.5 g NiCl_2_, 51 g agar powder (Agar‐Agar Kobe I, Carl Roth GmbH + Co. KG, Karlsruhe, Germany), 5.9 g NaCl and 0.4 g sodium azide). Active detuning for each loop was adjusted by the variable inductor L and measured as $S_{21}$ with a double‐probe by determining the difference between the loop's tuned and detuned states. For fine adjustments of the active detuning, a small solenoid “RF sniffer” probe was used with an $S_{11}$ measurement.

Preamplifier decoupling was assessed by changes in the double‐probe $S_{21}$ measurements, where each loop element was terminated with 50 Ω and the input impedance of the preamplifier, respectively. The unloaded‐to‐loaded coil quality factor ratio (*Q*_U_/*Q*_L_) of a representative 50 mm diameter loop within the populated but detuned coil array was measured with the phantom as a load using the $S_{21}$ double‐probe method.^52^ The circularly polarized Tx birdcage coil was tuned to the Larmor frequency under loaded condition using the end‐ring capacitors $C_{\text{ER}}$ and matched to 50 Ω with both the detuned Rx coil array and the ^19^F field camera probes present. Active tuning was realized with two PIN diodes $D_{Tx}$ per rung.

### 2.3 MRI data acquisition and analysis

MRI data were acquired on the 3 T Connectome 2.0 head scanner (MAGNETOM Connectom.X, Siemens Healthineers AG, Forchheim, Germany) with a $G_{\max}$ of 500 mT/m and an $SR_{\max}$ of 600 T/m/s. All experiments involving human tissue were approved by the Institutional Review Board at Massachusetts General Hospital.

The ex vivo coil imaging performance was compared with a 72‐channel in vivo head coil.^31^ Pixel‐wise SNR maps were obtained from a proton density‐weighted gradient‐echo sequence (repetition time (TR) = 200 ms, echo time (TE) = 4.8 ms, flip angle (F) = 15°, matrix (M) = 192 × 192, field of view (FOV) = 256 mm × 256 mm, slice thickness = 6 mm, bandwidth (BW) = 200 Hz/pixel, averages (A) = 6). Data for obtaining complex noise covariances and noise correlations were acquired using the same gradient‐echo sequence but without RF excitation or averaging. The SNR maps were computed using the method from Kellman et al.^53^ and normalized with an actual flip angle correction to account for differences in transmit coils. The actual flip angles were calculated following the double‐angle method^54^ with a gradient echo sequence.

The encoding capabilities of the ex vivo coil array were evaluated using reconstructed SENSE g‐factor maps^55^ with both 1D and 2D acceleration schemes.

**FIGURE 3:**
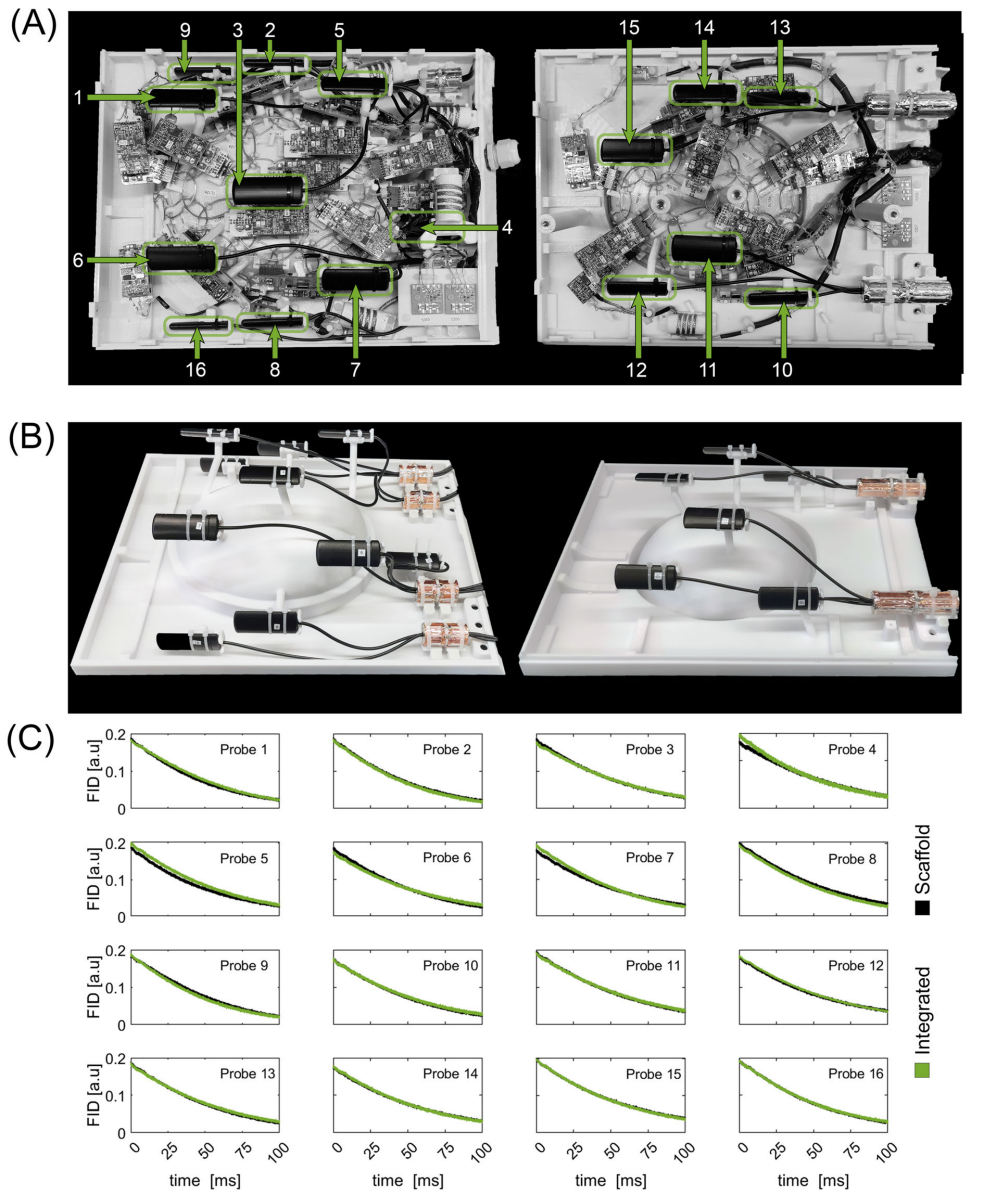
(A) Field Probes integrated in the receive coil. (B) Scaffold for field probes without receive coil, with same probe positions as the integrated version. (C) Free induction decay signal of the 16 field probes on the test scaffold without the receive coil surrounding (black) and integrated in the receive coil (green). The FID data was obtained by measuring 10 times and averaging. The probe lifetimes were nearly unaffected by the receive coil.

It is also crucial that the presence of the installed ^19^F field camera system does not impact the imaging performance of the Rx coil array. Therefore, we conducted the SNR measurements both before and after the installation of the field camera. Given the sensitivity of the ^19^F field camera probes to local field disturbances, such as susceptibility modulations or RF interference from the surrounding Rx coil electronics, we compared the free induction decay (FID) lifetime performance of the field probes with and without the fully assembled ex vivo coil array present. To ensure a consistence‐matched probe configuration of the ex vivo coil, an isolated scaffold field camera probe holder was constructed (Figure 3B).

To evaluate the ex vivo coil assembly's capability to provide temperature‐stabilized, long‐period dMRI scans, we conducted a 13‐hour diffusion scan with the forced‐air flow system activated (3D multi shot EPI sequence, shots: 4, b=0 and 4000 and 10000 s/mm^2^, $SR_{\max}$ = 435 T/m/s, $G_{\max}$ = 485 mT/m, directions = 35, TR = 500 ms, TE = 58 ms, number of slices = 20, voxel size = 0.9 mm iso, M = 230 × 230, FOV = 200 × 200 mm, number of scans = 17, total acquisition time = 13.04 h, BW = 1672 Hz/pixel). This experiment was then repeated without any air exchange inside the coil. For each measurement, we recorded the temperature. Both time‐series data sets were analyzed for mean diffusivity (MD) and mean kurtosis (MK).

Additional diffusion imaging was performed using a 3D EPI sequence that was modified with triggers at the beginning of the image readout to perform field monitoring. Acquisition parameters were: number of shots = 4, b = 0, 4000 (32 directions), and 10000 (48 directions) s/mm^2^, $SR_{\max}$ = 410 T/m/s, $G_{\max}$ = 498 mT/m, TR = 500 ms, TE = 53 ms, one slab, number of slices per slab = 176 (whole brain coverage), voxel size = 0.95 mm iso, M = 180 × 180, FOV = 170 × 170 mm, total acquisition time = 9 hours, Partial Fourier = 6/8, BW = 1736 Hz/pixel. The acquisition utilized both field monitoring and temperature stabilization. The images were reconstructed with a SENSE‐based reconstruction framework with the image encoding matrix that includes a three‐order spherical harmonic model for phase evolution.^36^ Coil sensitivities were estimated from a GRE sequence acquisition with the algorithm ESPIRit^56^ For additional details on $B_{0}$‐eddy current compensation removal, we refer the reader to.^36^ For the tractography analysis, all b‐shells (b = 4000 and 10 000 s/mm^2^) were concatenated. Further data preprocessing included MP‐PCA denoising with Rician floor correction^57^ and Gibbs ringing artifact removal.^58^ Brain extraction was performed on the temporal mean image (b = 4000) using SynthStrip,^59^ followed by white matter (WM) segmentation using SPM12.^60^ Fiber orientation distributions (fODFs) were reconstructed using multishell multitissue constrained spherical deconvolution.^61^, ^62^ Probabilistic tractography was performed using five random seed points per WM voxel, with maximum track length of 150 mm and FA termination threshold of 0.15. The resulting fODFs and tractography maps were visualized using mrview^63^ and rendered in 3D using DSI‐studio.^64^

## 3 RESULTS

The measured coil quality factor ratio (*Q*_U_/*Q*_L_) obtained of a 50‐mm loop element in the populated array was 180/77 = 2.34, demonstrating that noise is almost equally shared between the brain sample and coil, with slightly sample noise dominated regime. Preamplifier decoupling was established with an average value of 20 dB, ranging from 17 to 24 dB across the coil array. Coil elements adhered an average isolation between the tuned and detuned state of 38 dB, provided by the active detuning trap circuit.

**FIGURE 4:**
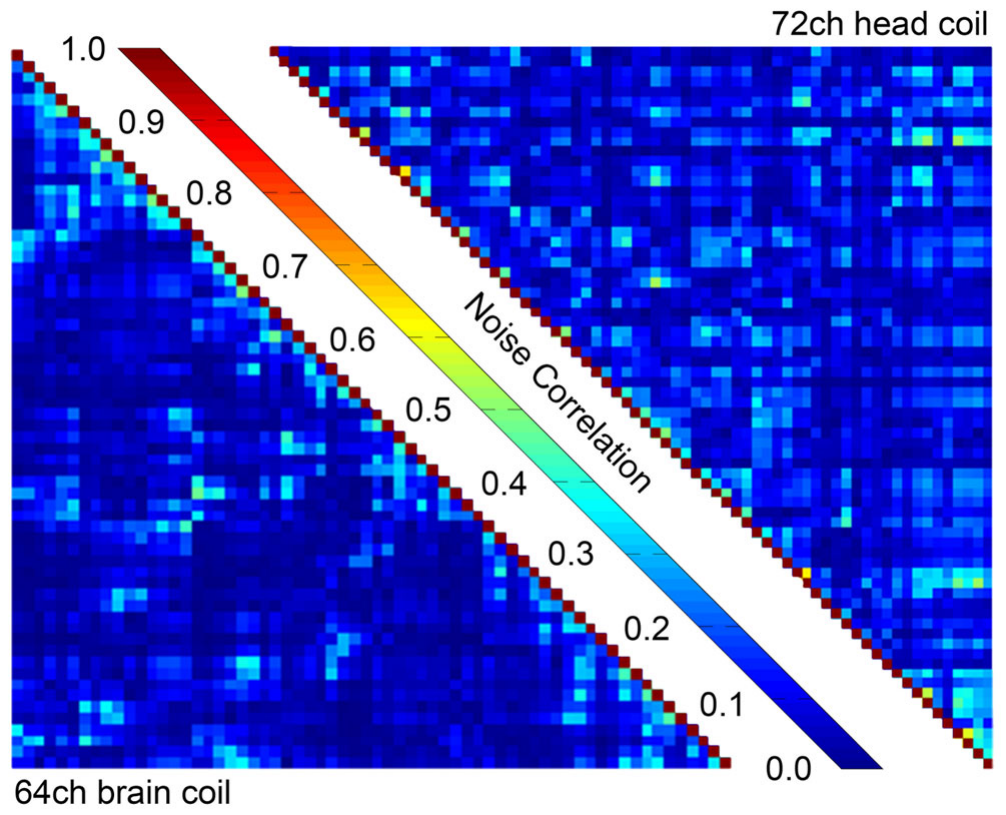
Noise correlation matrix of the developed coil in contrast to the 72‐channel head coil. The developed coil showed lower noise correlation in minimum, mean and maximum values.

Figure 4 shows the comparison of the noise correlation matrix from the ex vivo coil against the 72‐channel Connectome 2.0 in vivo head coil, when both coils were loaded with the brain‐shaped agar phantom. For the constructed ex vivo coil, the minimum, mean, and maximum noise correlation values were 0.05%, 8.94%, and 49.64%, respectively. For the in vivo head coil, these values were measured to be 0.23%, 11.98%, and 64.50%.

**FIGURE 5:**
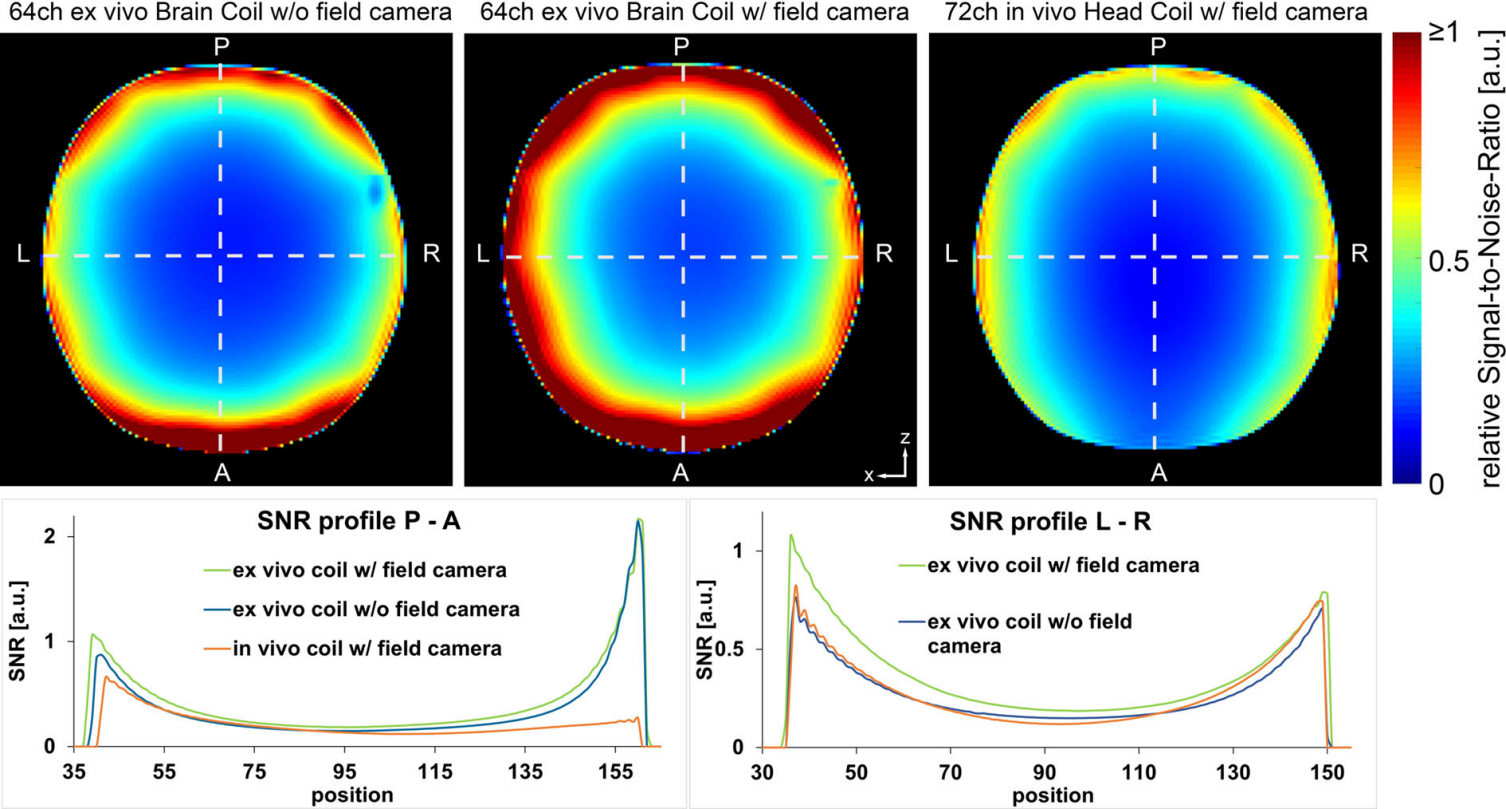
Comparison of flip‐angle‐corrected signal‐to‐noise‐ratio in a transversal (anatomical axis) slice. Top: SNR maps obtained from the ex vivo coil array without the field camera (top left), with the field camera (top middle), and the 72‐channel Connectome head coil (top right). Bottom: SNR profiles extracted along the dashed lines in posterior‐anterior (P‐A) and left‐right (L‐R) directions (anatomical axes of the brain) in the bottom. normalized to 1. The RF performance of the ex vivo coil array was optimized with the field camera present, contributing to its optimal SNR performance in this configuration.

In Figure 5, the flip‐angle‐corrected SNR is compared under three conditions: (A) the constructed brain coil without the ^19^F field probes present, (B) the same coil with integrated ^19^F field probes, and (C) the 72‐channel in vivo head coil. The incorporation of the field probes resulted in a 32% higher SNR compared with the configuration without them. When comparing the 64‐channel ex vivo coil with the 72‐channel head coil (both equipped with integrated field probes), the constructed ex vivo brain coil outperformed the head coil by 73% in SNR in the whole representative transversal slice. At the phantom's periphery, the SNR improvement was measured at 81%, and even in the center, an SNR gain of 48% was obtained. Further SNR comparisons are shown in Figure S5.

**FIGURE 6:**
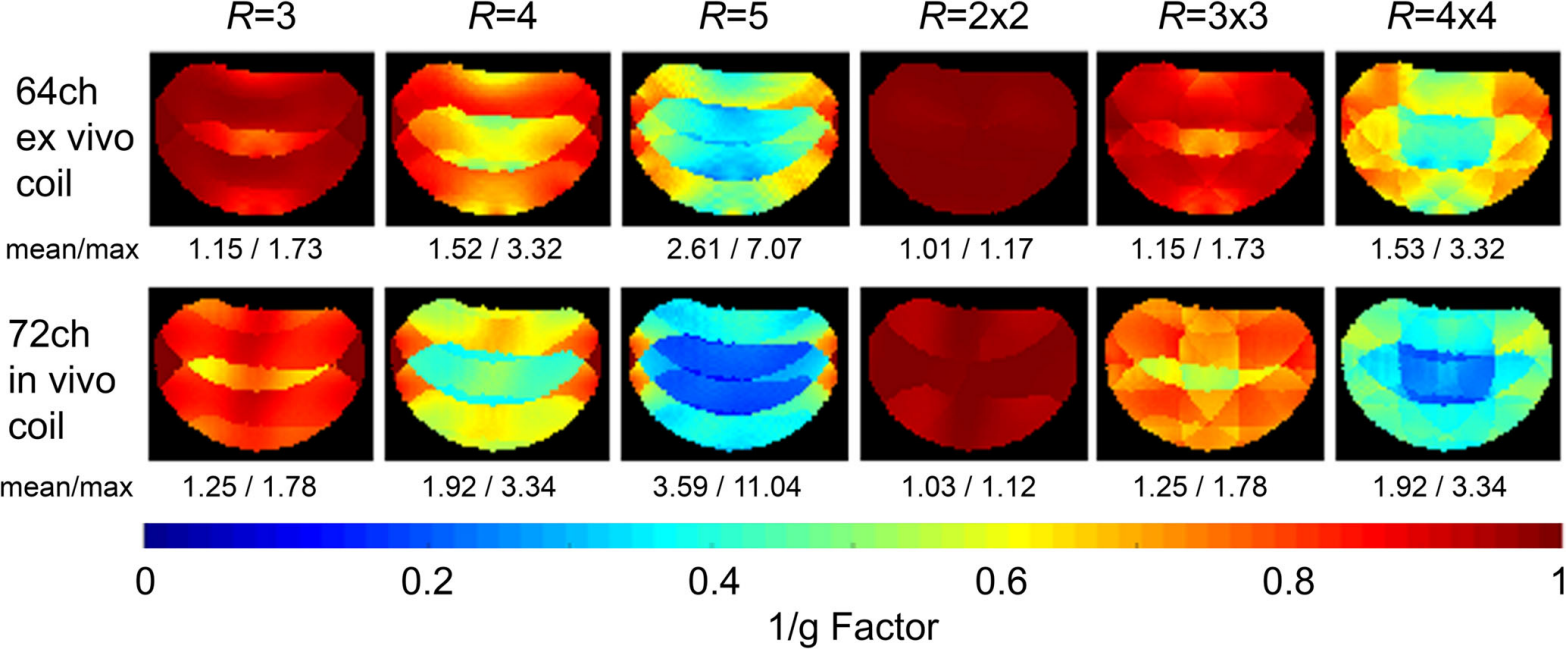
Inverse g‐factor maps of the 64‐channel ex vivo brain coil and the 72‐channel in vivo head coil. Both coils were equipped with the integrated field probes. The ex vivo coil slightly outperformed the in vivo head coil. The values below each map show the corresponding noninverted mean g‐factor and maximum g‐factor values of the entire representative slice.

When comparing acceleration capabilities between both coils (Figure 6), the 64‐channel ex vivo coil moderately outperformed the in vivo head coil in terms of both mean and maximum g‐factor values for 1D and 2D acceleration. For instance, with a reduction factor of 6, the average g‐factor of the ex vivo coil was 33% lower, and the maximum g‐factor was 46% lower in comparison with the in vivo head coil.

**FIGURE 7:**
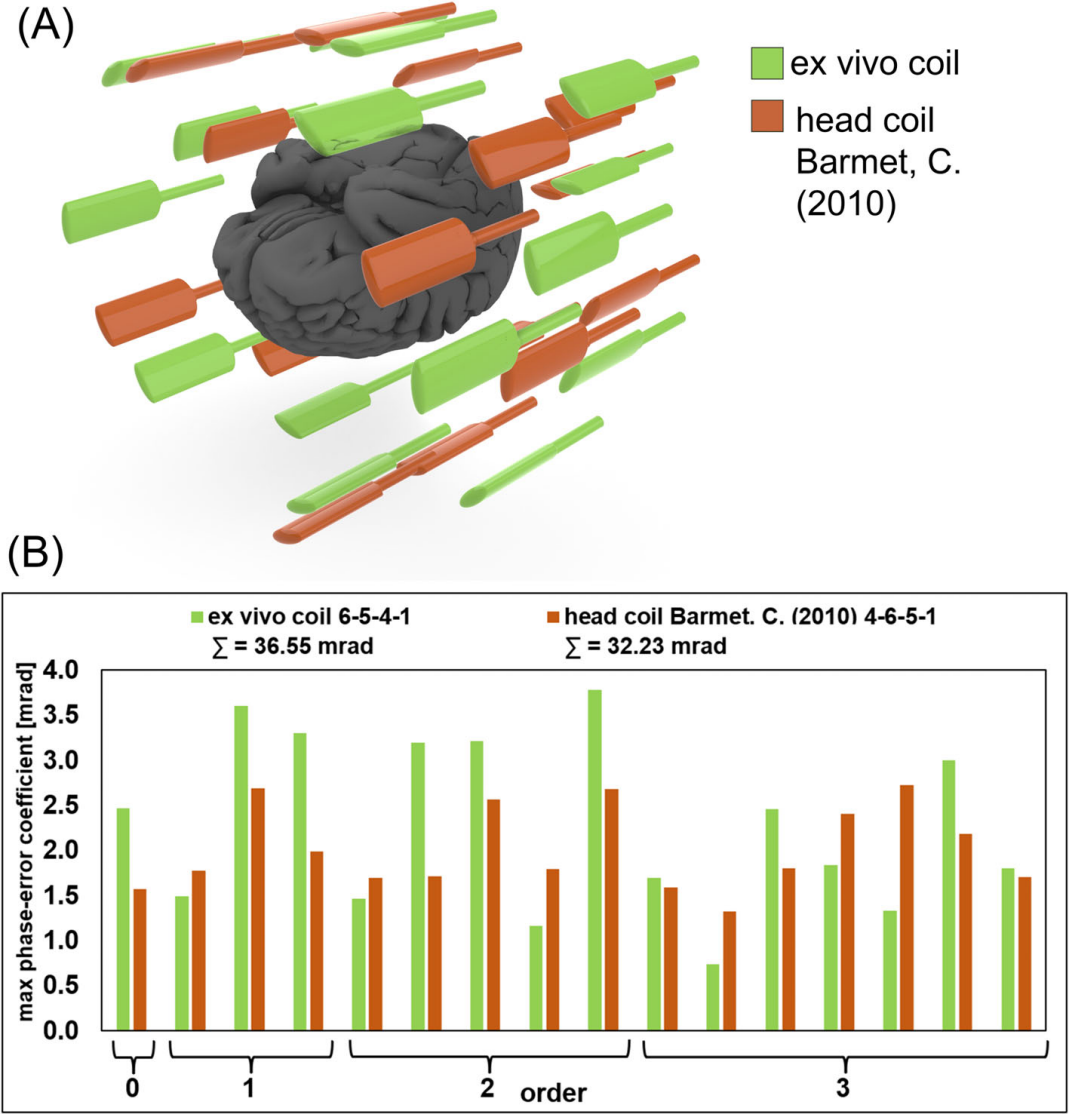
Comparison of the field probe arrangement between the developed ex vivo coil (green) and a reference head coil (orange) of Barmet et al.^25^, ^39^ (A) Geometric arrangement of both coils' probe configurations around the 3D brain model. (B) Comparison of the simulated maximum phase error for each spatial basis function of the spherical harmonics. The accumulated maximum phase error for the ex vivo coil was identified as 36.55 mrad, compared with accumulated value of 32.23 mrad for the reference head coil.^25^, ^39^

Figure 7 presents the analysis of the ^19^F field probe configuration, comparing it with well‐established coordinates from the literature^25^, ^39^ for MRI in vivo head coils. The accumulated total maximum phase error for the implemented distribution was calculated to be 36.55 mrad, while probe coordinates from the literature resulted in an accumulated value of 32.23 mrad. Thus, our final implemented configuration showed 4.32 mrad (11.8%) higher maximum phase error when compared with the literature's reference configurations. Figure S6 shows the computed maximum phase error of four additional probe configurations that represent key stages in our iterative optimization process. These configurations demonstrate the gradual improvement in performance achieved throughout our optimization efforts. While all probe positions were geometrically optimized to provide sufficient clearance to nearby RF components and materials, most alternative arrangements showed substantial higher maximum phase errors. One configuration with a more omni‐directional probe distribution performed comparably with our final implemented solution, but presented significant challenges for practical implementation due to cable routing constraints.

The FID lifetimes of the individual ^19^F field probes showed nearly identical decay profiles in both cases, whether the ^19^F field camera was integrated into the ex vivo coil array or used as a standalone installation on the scaffold probe holder (Figure 3). In both setups, the average lifetime of the ^19^F field probe signal (1/e ≈ 37% of maximum value) was identical at 56 ms. In the integrated setup, all probe lifetimes ranged from 47 to 63 ms, while in the standalone setup, the range was nearly identical at 48 to 62 ms.

**FIGURE 8:**
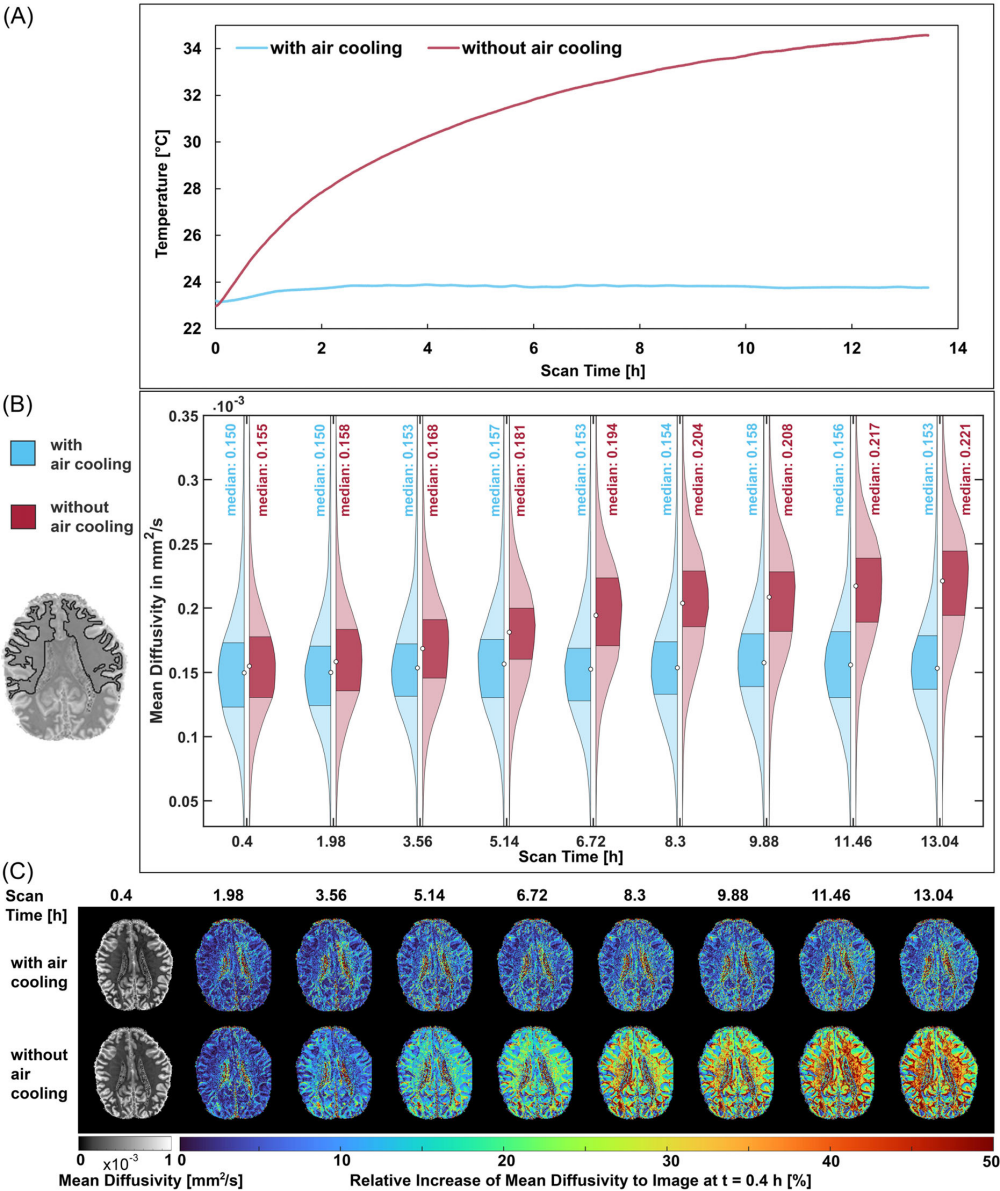
dMRI measurement series with and without forced air cooling, each over 13.04 hours (A) Temperature measurement with the two probes at the sample. (B) Violin plots of mean diffusivity values at nine time points in ROI of white matter. (C) Change in mean diffusivity relative to a reference image at 0.4 h scan time.

Figure 8 demonstrates the impact of connecting the coil housing to an external air blower, which facilitates the continuous exchange of air within the coil. In the absence of constant air exchange, the temperature of the ex vivo brain sample exhibited a gradual increase of 11.6°C over the course of a 13‐h dMRI scan. When active air exchange was used, an initial temperature rise of 0.75°C was observed within the first 2 h, plateauing to an equilibrium temperature for the remainder of the scan. Concurrently, a substantial increase of 22% in the mean diffusivity values in the white matter was measured during the 11.6°C temperature increase, while the mean kurtosis decreased considerably by 19% within the same temperature rise (Figure S7). With air‐flow cooling, there was only an increase of 2% in mean diffusivity and a decrease in mean kurtosis of 0.35%.

The reason for generally high values in the ventricles is due to the nature of pixel variation in this region. Unlike white and gray matter, the pixels within the ventricles exhibit more variability from pixel to pixel. During the imaging process, there is a chance of pixel displacement. This results in higher pixel‐to‐pixel deviation, although the true variability is not as pronounced as it appears.

**FIGURE 9:**
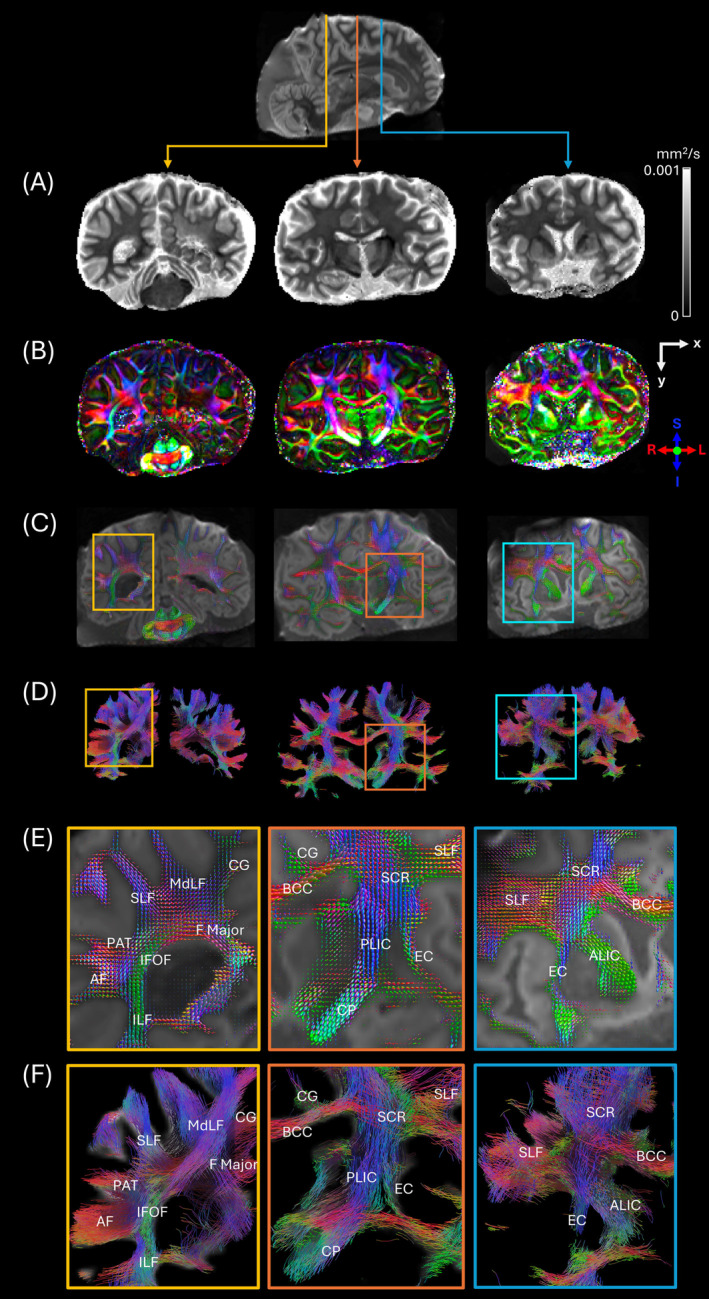
High‐resolution dMRI measurement with field monitoring. Each column shows (A) mean diffusivity (MD), (B) fractional anisotropy (FA), (C) fiber orientation density function (fODF), and (D) tractography from each coronal (anatomical axis) slice. In (C), fODF rendered using MRtrix3 is superimposed to b = 0 image. In (D), tractography is rendered in DSI studio. (E) and (F) represent the regions highlighted with colored boxes in fODF and tractgraphy, respectively. AF = arcuate fasciculus; ALIC = anterior lib of internal capsule; BCC = body of corpus callosum; CG = cingulum; CP = cerebral peduncle; EC = external capsule; F Major = forceps major; IFOF = inferior fronto‐occipital fasciculus; ILF = inferior longitudinal fasciculus; MdLF = middle longitudinal fasciculus; PAT = parietal aslant tract; PLIC = posterior limb of internal capsule; SCR = superior corona radiata; SLF = superior longitudinal fasciculus.

Mean diffusivity, fractional anisotropy, fiber orientation distribution functions, and tractography were successfully reconstructed from the field camera‐corrected diffusion MRI data in three different brain slabs (Figure 9A‐D). Multiple major white matter tracts were clearly identifiable in magnified captures from both the fODFs and tractography results (Figure 9E,F), including the arcuate fasciculus, anterior limb of internal capsule, body of corpus callosum, cingulum, cerebral peduncle, external capsule, forceps major, inferior fronto‐occipital fasciculus, inferior longitudinal fasciculus, middle longitudinal fasciculus, parietal aslant tract, posterior limb of internal capsule, superior corona radiata, and superior longitudinal fasciculus.

## 4 DISCUSSION

This study addressed several technical challenges associated with strong diffusion‐sensitizing and high‐resolution ex vivo dMRI by developing a 64‐channel receive coil array integrated with field monitoring and temperature stabilization systems and a dedicated Tx birdcage coil. The work highlights three key improvements: enhanced SNR, reduced image artifacts, and stable temperature management, all critical for long scans of post‐mortem human brain specimens.

### 4.1 Receive coil design

Achieving high b‐value diffusion‐weighted ex vivo imaging with submillimeter resolution presents significant challenges due to its inherently SNR‐starved acquisition. To address this issue, we used a two‐pronged approach leveraging both intrinsic and external SNR enhancement strategies. The Connectome 2.0 scanner's advanced hardware capabilities, specifically its high gradient strength and slew rate, contributed to preserving intrinsic diffusion‐weighted SNR. Complementing the gradient coil's capabilities, we optimized the RF coil detector technology, which represents a critical external component in preserving SNR within the signal detection chain.

The ex vivo imaging context provided distinct SNR advantages. Our design capitalized on several key factors: (1) The fully enclosed configuration enabled omnidirectional signal reception, capturing signals from all regions of the specimen; (2) the dielectric properties of fixation media supported the use of smaller detector elements (approximately 50 mm diameter) while maintaining sample‐noise dominance; and (3) safety requirements for ex vivo imaging allowed us to omit certain components (e.g., fuses and passive detuning circuits) that typically degrade Q‐factor in in vivo applications. These combined advantages facilitated our anatomically conforming design with a favorable Q‐ratio of 2.34.

### 4.2 Field monitoring

The utilization of fast‐slewing, high‐gradient field strengths introduces significant challenges in diffusion image quality. These high‐performing gradients generate intense eddy currents and higher order Maxwell terms, resulting in image artifacts that scale with the strength of the diffusion‐encoding gradient pulses. While spatially linear eddy current fields can be mitigated through careful gradient pulse design, such as pre‐emphasis techniques, the higher order spatial eddy currents with multiple time constants, combined with nonlinear gradient Maxwell terms, present more complex challenges. These effects lead to spatially variable image distortions that compromise both geometry and resolution across the reconstructed image. The implementation of a 16‐channel field camera system has proven highly effective in mitigating these issues.^25^, ^26^, ^36^, ^65^, ^66^ The integration in our coil eliminates the need for separate calibration prescans, streamlining the imaging workflow while potentially improving field dynamics compensation accuracy.

The successful integration of the RF field camera into the 64‐channel ex vivo coil depended on three critical considerations. First, we ensured the field camera and receiver coil structure maintained nearly independent operation without negatively impacting each other. The minor SNR decrease when the field camera was removed is likely because the array coil was tuned and matched with the field camera in place. This suggests that the field camera interacts with the Rx array layer, and thus the final coil tuning and matching process must be performed when the field camera probes are present and fully assembled. This minimal interaction could potentially originate from the ex vivo coil elements having an overall high loaded Q factor of 77. These loop elements, which feature smaller dimensions and fewer Q‐spoiling safety components compared with typical in vivo coils, are consequently more susceptible to electromagnetic interactions with nearby components. Second, we evaluated field camera function by measuring FID lifetimes in both the fully integrated assembly and when isolated on a scaffold, maintaining identical probe positioning. The lifetime is critical to maximize the duration of continuous monitoring within one excitation.^32^ The absence of significant FID performance differences validates the integrated design's robustness.

Lastly, the complete determination of a third‐order spherical harmonics expansion of the target volume enabled us to evaluate different field probe positioning configurations. While omni‐directional probe distributions are theoretically appealing for ex vivo brains—unlike in vivo head imaging where neck anatomy restricts probe placement—our iterative optimization process revealed practical limitations to implementing such arrangements. Despite the enhanced positioning freedom offered by ex vivo specimens, we encountered significant limitations related to cable routing, RF component clearance requirements, and the need for robust mechanical stability, all of which guided our final configuration selection. Notably, the 4.32 mrad‐difference in total accumulated maximum phase error between our implemented configuration and established reference values from the literature,^25^, ^39^ does not appear to compromise the functionality of our field‐monitored diffusion MRI acquisitions. Furthermore, this systematic evaluation confirms that field probe arrangements can be effectively adapted to specialized coil designs while preserving essential monitoring capabilities.

### 4.3 Temperature stabilization

Temperature‐related drifts in quantitative dMRI measurements are well‐documented, as T_1_ and diffusivity depend heavily on brain sample temperature.^42^, ^67^, ^68^ Our ex vivo coil system also addressed thermal management during prolonged dMRI scans through six fiber optic temperature probes and forced air circulation. Air cooling was chosen deliberately over alternatives like water beds for its noninterference with MRI signals. The simple but efficient approach for temperature stabilization benefits from the easy handling due to its integration into the coil system and removability of the hoses and fan. The significance of this cooling system became evident during 13‐hour postmortem scans, which revealed temperature increases up to 11.6°C without temperature stabilization.

While our current open‐loop forced air cooling system substantially mitigates temperature increases, we still observed a 2% increase in mean diffusivity and a 0.35% decrease in mean kurtosis over 13 hours. These subtle but measurable changes highlight the need for further refinement of thermal management strategies. This suggests the implementation of a closed‐loop temperature control system that incorporates a chiller unit to regulate input air temperature precisely. Such advancement would enable more responsive temperature adjustments and greater stability during extended imaging sessions. The ex vivo coil is already outfitted with strategically placed temperature sensors, allowing the technical implementation with constant temperature feedback data for closed‐loop monitoring and control, without requiring any hardware modifications.

### 4.4 Imaging performance

The 64‐channel ex vivo coil demonstrated substantial improvements over the 72‐channel in vivo head coil through enhanced SNR and encoding capabilities. Despite having fewer channels, the ex vivo coil achieved higher SNR, which can be attributed to several key factors: The adaptation of the receive circuitry to the specific dielectric properties of ex vivo brain tissue and the strategically designed anatomical conformal coil layout, tailored to encompass a whole brain specimen. The fully enclosed design of the ex vivo coil enabled omni‐directional signal reception, although it should be noted that detector loop elements aligned along the z‐axis may have limited efficiency in detecting transverse magnetization component. In contrast, typical in vivo head coils are not fully enclosed due to patient positioning requirements, which results in suboptimal signal reception for ex vivo brain samples.

The close‐fitting design and smaller detector elements enhance spatial signal intensity modulation within the imaging volume, thereby improving both SENSE‐based unaliasing and GRAPPA‐like spatial harmonics synthesis, characteristics that facilitate accelerated imaging.

The high‐resolution diffusion‐weighted images underscore the optimal interplay between the acquisition quality of the 64‐channel receive array, the correction capabilities provided by integrated field monitoring, and the enhanced fidelity achieved through temperature stabilization. Together, these factors contribute to exceptionally reliable and high‐fidelity, high‐resolution diffusion imaging, demonstrating the system's efficacy for advanced ex vivo brain investigations under demanding acquisition conditions.

## 5 CONCLUSION

We successfully designed, constructed, and evaluated a 64‐channel receive array coil as well as a dedicated Tx birdcage coil, and optimized both for high‐resolution, high‐fidelity diffusion‐weighted imaging of whole human ex vivo brains. The anatomically conformed design and precise RF optimizations provided high SNR, making it well‐suited for high b‐value dMRI acquisitions. Our comprehensive artifact mitigation strategy incorporated low eddy current shield design, an integrated field camera, and temperature stabilization system. When combined with the Connectome 2.0 scanner's high gradient strength, the ex vivo coil system enables unprecedented submillimeter‐resolution diffusion imaging of the human ex vivo brain.

## CONFLICT OF INTEREST STATEMENT

The authors declare no potential conflict of interests.

## Supporting information


**Data S1.** Supporting Information.

## Data Availability

The data that support findings of this study are openly available in github at https://github.com/keyarray/connectexvivocoil.^69^ This includes 3D‐CAD files for all mechanical coil parts, including the validation phantom and Gerber files of the PCB circuits.

## References

[1] McNab JA , Edlow BL , Witzel T , et al. The human connectome project and beyond: initial applications of 300 mT/m gradients. Neuroimage. 2013;80:234‐245. doi:10.1016/j.neuroimage.2013.05.074 23711537 PMC3812060

[2] Setsompop K , Kimmlingen R , Eberlein E , et al. Pushing the limits of in vivo diffusion MRI for the human connectome project. Neuroimage. 2013;80:220‐233. doi:10.1016/j.neuroimage.2013.05.078 23707579 PMC3725309

[3] Assaf Y , Blumenfeld‐Katzir T , Yovel Y , Basser PJ . Axcaliber: a method for measuring axon diameter distribution from diffusion MRI. Magn Reson Med. 2008;59:1347‐1354. doi:10.1002/mrm.21577 18506799 PMC4667732

[4] Beaujoin J , Palomero‐Gallagher N , Boumezbeur F , et al. Post‐mortem inference of the human hippocampal connectivity and microstructure using ultra‐high field diffusion MRI at 11.7 T. Brain Struct Funct. 2018;223:2157‐2179. doi:10.1007/s00429-018-1617-1 29387938 PMC5968081

[5] Schilling KG , Howard AFD , Grussu F , et al. Considerations and recommendations from the ISMRM diffusion study Group for Preclinical Diffusion MRI: part 3—ex vivo imaging: data processing, comparisons with microscopy, and Tractography. Magn Reson Med. 2025;93:2561‐2582. doi:10.1002/mrm.30424 40008460 PMC11971500

[6] Scholz A , Etzel R , May MW , et al. A 48‐channel receive Array coil for mesoscopic diffusion‐weighted MRI of ex vivo human brain on the 3 T connectome scanner. Neuroimage. 2021;238:118256. doi:10.1016/j.neuroimage.2021.118256 34118399 PMC8439104

[7] Ramos‐Llordén G , Maffei C , Tian Q , et al. Ex‐vivo whole human brain high b‐value diffusion MRI at 550 Micron with a 3T Connectom scanner. In: Proceedings of the 29th Annual Meeting of ISMRM. Virtual Meeting. International Society for Magnetic Resonance in Medicine; 2021:300.

[8] Ramos‐Llordén G , Lobos RA , Kim TH , et al. High‐Fidelity, high‐spatial‐resolution diffusion magnetic resonance imaging of ex vivo whole human brain at ultra‐high gradient strength with structured low‐rank Echo‐planar imaging ghost correction. NMR Biomed. 2023;36:e4831. doi:10.1002/nbm.4831 36106429 PMC9883835

[9] Augustinack JC , Helmer K , Huber KE , Kakunoori S , Zöllei L , Fischl B . Direct visualization of the Perforant pathway in the human brain with ex vivo diffusion tensor imaging. Front Hum Neurosci. 2010;4:42. doi:10.3389/fnhum.2010.00042 20577631 PMC2889718

[10] Fritz FJ , Sengupta S , Harms RL , Tse DH , Poser BA , Roebroeck A . Ultra‐high resolution and multi‐Shell diffusion MRI of intact ex vivo human brains using kT‐dSTEAM at 9.4T. Neuroimage. 2019;202:116087. doi:10.1016/j.neuroimage.2019.116087 31408716

[11] Miller KL , Stagg CJ , Douaud G , et al. Diffusion imaging of whole, post‐mortem human brains on a clinical MRI scanner. Neuroimage. 2011;57:167‐181. doi:10.1016/j.neuroimage.2011.03.070 21473920 PMC3115068

[12] Jones R , Grisot G , Augustinack J , et al. Insight into the fundamental trade‐offs of diffusion MRI from polarization‐sensitive optical coherence tomography in ex vivo human brain. Neuroimage. 2020;214:116704. doi:10.1016/j.neuroimage.2020.116704 32151760 PMC8488979

[13] Boonstra JT , Michielse S , Roebroeck A , Temel Y , Jahanshahi A . Dedicated container for Postmortem human brain ultra‐high field magnetic resonance imaging. Neuroimage. 2021;235:118010. doi:10.1016/j.neuroimage.2021.118010 33819610

[14] Edlow BL , Mareyam A , Horn A , et al. 7 tesla MRI of the ex vivo human brain at 100 Micron resolution. Sci Data. 2019;6:244. doi:10.1038/s41597-019-0254-8 31666530 PMC6821740

[15] Roebroeck A , Sengupta S , Bastiani M , et al. High resolution MRI neuroanatomy in whole human brains post mortem with a specialized 9.4T RF‐coil. Proc Annu Meet Organ Hum Brain Mapp. Honolulu, USA. Organization for Human Brain Mapping; 2015:1856. doi:10.13140/RG.2.2.21380.07041

[16] Gruber B , Keil B , Witzel T , Nummenmaa A , Wald L . A 60‐channel ex‐vivo brain‐slice coil Array for 3T imaging. Proceedings of the 23rd Annual Meeting of ISMRM. Milan, Italy. International Society for Magnetic Resonance in Medicine; 2014:4885.

[17] Sengupta S , Fritz FJ , Harms RL , et al. High resolution anatomical and quantitative MRI of the entire human occipital lobe ex vivo at 9.4T. Neuroimage. 2018;168:162‐171. doi:10.1016/j.neuroimage.2017.03.039 28336427 PMC5862655

[18] Plantinga BR , Roebroeck A , Kemper VG , et al. Ultra‐high field MRI post mortem structural connectivity of the human subthalamic nucleus, substantia Nigra, and Globus Pallidus. Front Neuroanat. 2016;10:10. 27378864 10.3389/fnana.2016.00066 PMC4909758

[19] Pallebage‐Gamarallage M , Foxley S , Menke RAL , et al. Dissecting the pathobiology of altered MRI signal in amyotrophic lateral sclerosis: a post mortem whole brain sampling strategy for the integration of ultra‐high‐field MRI and quantitative neuropathology. BMC Neurosci. 2018;19:11. doi:10.1186/s12868-018-0416-1 29529995 PMC5848544

[20] D'Arceuil HE , Westmoreland S , de Crespigny AJ . An approach to high resolution diffusion tensor imaging in fixed primate brain. Neuroimage. 2007;35:553‐565. doi:10.1016/j.neuroimage.2006.12.028 17292630

[21] Huang SY , Witzel T , Keil B , et al. Connectome 2.0: developing the next‐generation ultra‐high gradient strength human MRI scanner for bridging studies of the micro‐, Meso‐ and macro‐connectome. Neuroimage. 2021;243:118530. doi:10.1016/j.neuroimage.2021.118530 34464739 PMC8863543

[22] Ramos‐Llordén G , Dietz P , Davids M , et al. Connectome 2.0: performance evaluation and initial in vivo human brain diffusion MRI results. Proceedings of the 32nd Annual Meeting of ISMRM. Singapore. International Society for Magnetic Resonance in Medicine; 2024:901.

[23] Yendiki A , Aggarwal M , Axer M , Howard AFD , van Walsum AMC , Haber SN . Post mortem mapping of connectional anatomy for the validation of diffusion MRI. Neuroimage. 2022;256:119146. doi:10.1016/j.neuroimage.2022.119146 35346838 PMC9832921

[24] Roebroeck A , Miller KL , Aggarwal M . Ex vivo diffusion MRI of the human brain: technical challenges and recent advances. NMR Biomed. 2019;32:e3941. doi:10.1002/nbm.3941 29863793 PMC6492287

[25] Wilm BJ , Nagy Z , Barmet C , et al. Diffusion MRI with concurrent magnetic field monitoring. Magn Reson Med. 2015;74:925‐933. doi:10.1002/mrm.25827 26183218

[26] Gilbert KM , Dubovan PI , Gati JS , Menon RS , Baron CA . Integration of an RF coil and commercial field camera for ultrahigh‐field MRI. Magn Reson Med. 2022;87:2551‐2565. doi:10.1002/mrm.29130 34932225

[27] Valsamis JJ , Dubovan PI , Baron CA . Characterization and correction of time‐varying Eddy currents for diffusion MRI. Magn Reson Med. 2022;87:2209‐2223. doi:10.1002/mrm.29124 34894640

[28] Boesch C , Gruetter R , Martin E . Temporal and spatial analysis of fields generated by Eddy currents in superconducting magnets: optimization of corrections and quantitative characterization of magnet/gradient systems. Magn Reson Med. 1991;20:268‐284. doi:10.1002/mrm.1910200209 1775052

[29] Mahmutovic M , Scholz A , Kutscha N , et al. A 64‐channel brain Array coil with an integrated 16‐channel field monitoring system for 3T MRI. Proceedings of the 29th Annual Meeting of ISMRM. Virtual Meeting. International Society for Magnetic Resonance in Medicine; 2021:623.

[30] Brunner DO , Gross S , Schmid T , et al. Integration of field monitoring for neuroscientific applications ‐ SNR, acceleration and image integrity. Proceedings of the 27th Annual Meeting of ISMRM. Montreal, Canada. International Society for Magnetic Resonance in Medicine; 2019:1046.

[31] Mahmutovic M , Shrestha M , Ramos‐Llordén G , et al. A 72‐channel head coil with an integrated 16‐channel field camera for the 3T connectome 2.0 scanner. Proceedings of the 32nd Annual Meeting of ISMRM. Singapore. International Society for Magnetic Resonance in Medicine; 2024:1027.

[32] DeZanche N , Barmet C , Nordmeyer‐Massner JA , Pruessmann KP . NMR probes for measuring magnetic fields and field dynamics in MR systems. Magn Reson Med. 2008;60:176‐186. doi:10.1002/mrm.21624 18581363

[33] Barmet C , Zanche ND , Pruessmann KP . Spatiotemporal magnetic field monitoring for MR. Magn Reson Med. 2008;60:187‐197. doi:10.1002/mrm.21603 18581361

[34] Roméo F , Hoult DI . Magnet field profiling: analysis and correcting coil design. Magn Reson Med. 1984;1:44‐65. doi:10.1002/mrm.1910010107 6571436

[35] Vannesjo SJ , Wilm BJ , Duerst Y , et al. Retrospective correction of physiological field fluctuations in high‐field brain MRI using concurrent field monitoring. Magn Reson Med. 2015;73:1833‐1843. doi:10.1002/mrm.25303 24903278

[36] Ramos‐Llordén G , Park DJ , Kirsch JE , et al. Eddy current‐induced Artifact correction in high b‐value ex vivo human brain diffusion MRI with dynamic field monitoring. Magn Reson Med. 2024;91:541‐557. doi:10.1002/mrm.29873 37753621 PMC10842131

[37] Chu S , Gras V , Boulant N , Gunamony S . 16‐channel 11.7T transmit Array with integrated field probes. Proceedings of the 31st Annual Meeting of ISMRM. Toronto, Canada. International Society for Magnetic Resonance in Medicine; 2023:4589.

[38] Kennedy M , Lee Y , Nagy Z . An industrial design solution for integrating NMR magnetic field sensors into an MRI scanner. Magn Reson Med. 2018;80:833‐839. doi:10.1002/mrm.27055 29285786

[39] Barmet C , Wilm BJ , Pavan M , et al. Concurrent higher‐order field monitoring for routine head MRI: an integrated Heteronuclear setup. Proceedings of the 19th Annual Meeting of ISMRM. International Society for Magnetic Resonance in Medicine; 2010:216.

[40] Brunheim S , Mirkes C , Dietrich BE , et al. Replaceable field probe holder for the Nova coil on a 7 tesla Siemens scanner. Proceedings of the 28th Annual Meeting of ISMRM. Vitrual Meeting. International Society for Magnetic Resonance in Medicine; 2020:3389.

[41] Reischauer C , Staempfli P , Jaermann T , Boesiger P . Construction of a temperature‐controlled diffusion phantom for quality control of diffusion measurements. J Magn Reson Imaging. 2009;29:692‐698. doi:10.1002/jmri.21665 19243053

[42] Rieger SW , Hess A , Ji Y , et al. A temperature‐controlled cooling system for accurate quantitative post‐mortem MRI. Magn Reson Med. 2023;90:2643‐2652. doi:10.1002/mrm.29816 37529979 PMC10952464

[43] Kumar A , Edelstein WA , Bottomley PA . Noise figure limits for circular loop MR coils. Magn Reson Med. 2009;61:1201‐1209. doi:10.1002/mrm.21948 19253376 PMC2869245

[44] Reykowski A , Wright SM , Porter JR . Design of Matching Networks for low noise preamplifiers. Magn Reson Med. 1995;33:848‐852. doi:10.1002/mrm.1910330617 7651124

[45] Roemer PB , Edelstein WA , Hayes CE , Souza SP , Mueller OM . The NMR phased Array. Magn Reson Med. 1990;16:192‐225. doi:10.1002/mrm.1910160203 2266841

[46] Edelstein WA , Hardy CJ , Mueller OM . Electronic decoupling of surface‐coil receivers for NMR imaging and spectroscopy. J Magn Reson. 1986;67:156‐161. doi:10.1016/0022-2364(86)90421-X

[47] Keil B , Blau JN , Biber S , et al. A 64‐channel 3T Array coil for accelerated brain MRI. Magn Reson Med. 2013;70:248‐258. doi:10.1002/mrm.24427 22851312 PMC3538896

[48] Barmet C , Wilm BJ , Pavan M , Pruessmann KP . A third‐order field camera with microsecond resolution for MR system diagnostics. Proceedings of the 17th Annual Meeting of ISMRM. Honululu, USA. International Society for Magnetic Resonance in Medicine; 2009:781.

[49] Seeber D , Jevtic J , Menon A . Floating shield current suppression trap. Concepts Magn Reson B Magn Reson Eng. 2004;21B:26‐31. doi:10.1002/cmr.b.20008

[50] Ghotra A , Kosakowski HL , Takahashi A , et al. A size‐adaptive 32‐channel Array coil for awake infant neuroimaging at 3 tesla MRI. Magn Reson Med. 2021;86:1773‐1785. doi:10.1002/mrm.28791 33829546

[51] Friedman L , Glover GH . Report on a Multicenter fMRI quality assurance protocol. J Magn Reson Imaging. 2006;23:827‐839. doi:10.1002/jmri.20583 16649196

[52] Hoult DI . The NMR receiver: a description and analysis of design. Prog Nucl Magn Reson Spectrosc. 1978;12:41‐77. doi:10.1016/0079-6565(78)80002-8

[53] Kellman P , McVeigh ER . Image reconstruction in SNR units: a general method for SNR measurement. Magn Reson Med. 2005;54:1439‐1447. doi:10.1002/mrm.20713 16261576 PMC2570032

[54] Stollberger R , Wach P . Imaging of the active B1 field in vivo. Magn Reson Med. 1996;35:246‐251. doi:10.1002/mrm.1910350217 8622590

[55] Pruessmann KP , Weiger M , Scheidegger MB , Boesiger P . SENSE: sensitivity encoding for fast MRI. Magn Reson Med. 1999;42:952‐962. doi:10.1002/(SICI)1522-2594(199911)42:5¡952::AID-MRM16¿3.0.CO;2-S 10542355

[56] Uecker M , Lai P , Murphy MJ , et al. ESPIRiT—an eigenvalue approach to autocalibrating parallel MRI: where SENSE meets GRAPPA. Magn Reson Med. 2014;71:990‐1001. doi:10.1002/mrm.24751 23649942 PMC4142121

[57] Veraart J , Fieremans E , Novikov DS . Diffusion MRI noise mapping using random matrix theory. Magn Reson Med. 2016;76:1582‐1593. doi:10.1002/mrm.26059 26599599 PMC4879661

[58] Kellner E , Dhital B , Kiselev VG , Reisert M . Gibbs‐ringing Artifact removal based on local subvoxel‐shifts. Magn Reson Med. 2016;76:1574‐1581. doi:10.1002/mrm.26054 26745823

[59] Hoopes A , Mora JS , Dalca AV , Fischl B , Hoffmann M . SynthStrip: skull‐stripping for any brain image. Neuroimage. 2022;260:119474. doi:10.1016/j.neuroimage.2022.119474 35842095 PMC9465771

[60] Ashburner J , Friston KJ . Unified segmentation. Neuroimage. 2005;26:839‐851. doi:10.1016/j.neuroimage.2005.02.018 15955494

[61] Jeurissen B , Tournier JD , Dhollander T , Connelly A , Sijbers J . Multi‐tissue constrained spherical deconvolution for improved analysis of multi‐Shell diffusion MRI data. Neuroimage. 2014;103:411‐426. doi:10.1016/j.neuroimage.2014.07.061 25109526

[62] Dhollander T , Mito R , Raffelt D , Connelly A . Improved white matter response function estimation for 3‐tissue constrained spherical deconvolution. Proceedings of the 27th Annual Meeting of ISMRM. Montréal, Québec, Canada. International Society for Magnetic Resonance in Medicine; 2019:555.

[63] Tournier JD , Smith R , Raffelt D , et al. *MRtrix3*: a fast, flexible and open software framework for medical image processing and visualisation. Neuroimage. 2019;202:116137. doi:10.1016/j.neuroimage.2019.116137 31473352

[64] Yeh FC . Population‐based tract‐to‐region connectome of the human brain and its hierarchical topology. Nat Commun. 2022;13:4933. doi:10.1038/s41467-022-32595-4 35995773 PMC9395399

[65] Wilm BJ , Barmet C , Gross S , et al. Single‐shot spiral imaging enabled by an expanded encoding model: demonstration in diffusion MRI. Magn Reson Med. 2017;77:83‐91. doi:10.1002/mrm.26493 27770473

[66] Engel M , Kasper L , Barmet C , et al. Single‐Shot Spiral Imaging at 7 T. Magn Reson Med. 2018;80:1836‐1846. doi:10.1002/mrm.27176 29575161

[67] Bottomley PA , Foster TH , Argersinger RE , Pfeifer LM . A review of Normal tissue hydrogen NMR relaxation times and relaxation mechanisms from 1–100 MHz: dependence on tissue type, NMR frequency, temperature, species, excision, and age. Med Phys. 1984;11:425‐448. doi:10.1118/1.595535 6482839

[68] Birkl C , Langkammer C , Haybaeck J , et al. Temperature‐induced changes of magnetic resonance relaxation times in the human brain: a Postmortem study. Magn Reson Med. 2014;71:1575‐1580. doi:10.1002/mrm.24799 23716457

[69] Müller A . Github Repository. Accessed May 15, 2025. https://github.com/keyarray/connectexvivocoil

