# Supplementary material for: Strong gradients, cool performance: A 64‐channel array coil with concurrent field monitoring and thermal control for ex vivo diffusion‐weighted brain imaging using the 3T connectome 2.0 MRI scanner
Source: Magn Reson Med. 2025 Jun 8;94(5):2268–85. doi: 10.1002/mrm.30599 (PMC12393195; doi:10.1002/mrm.30599)
Supplement: Supplementary file 1 — Data S1. Supporting Information. [file MRM-94-2268-s001.pdf]

Supporting Information Figures

Strong Gradients, Cool Performance: A 64-channel array coil with concurrent field monitoring and thermal control for ex vivo diffusion-weighted brain imaging using the 3T Connectome 2.0 MRI Scanner

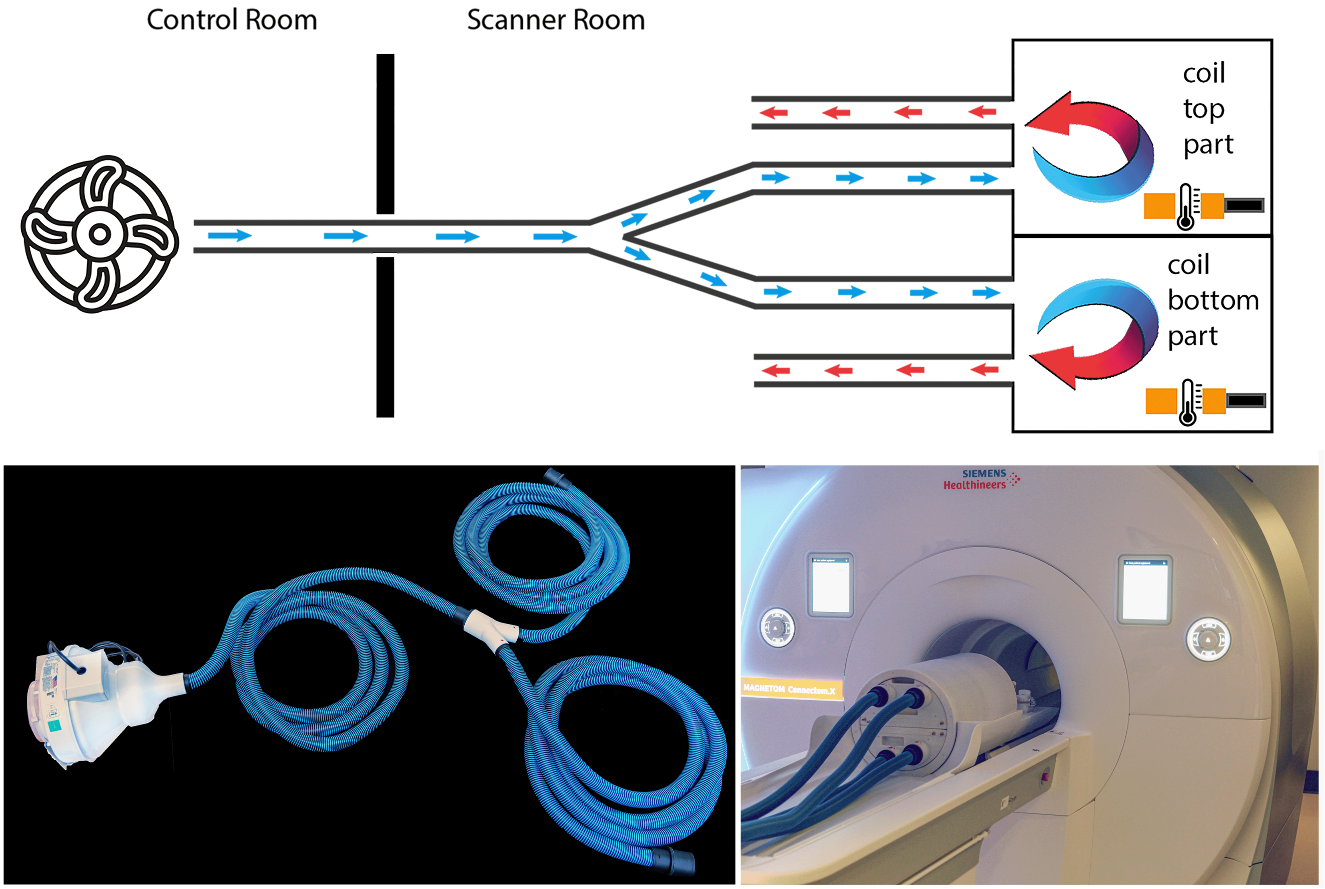

Figure S1: Assembly of the temperature stabilization system, consisting of a fan (left), air hoses for fresh room temperature air (blue) and warm “coil air” (red in outline) and six (three in each coil part) temperature probes (orange) inside the coil.

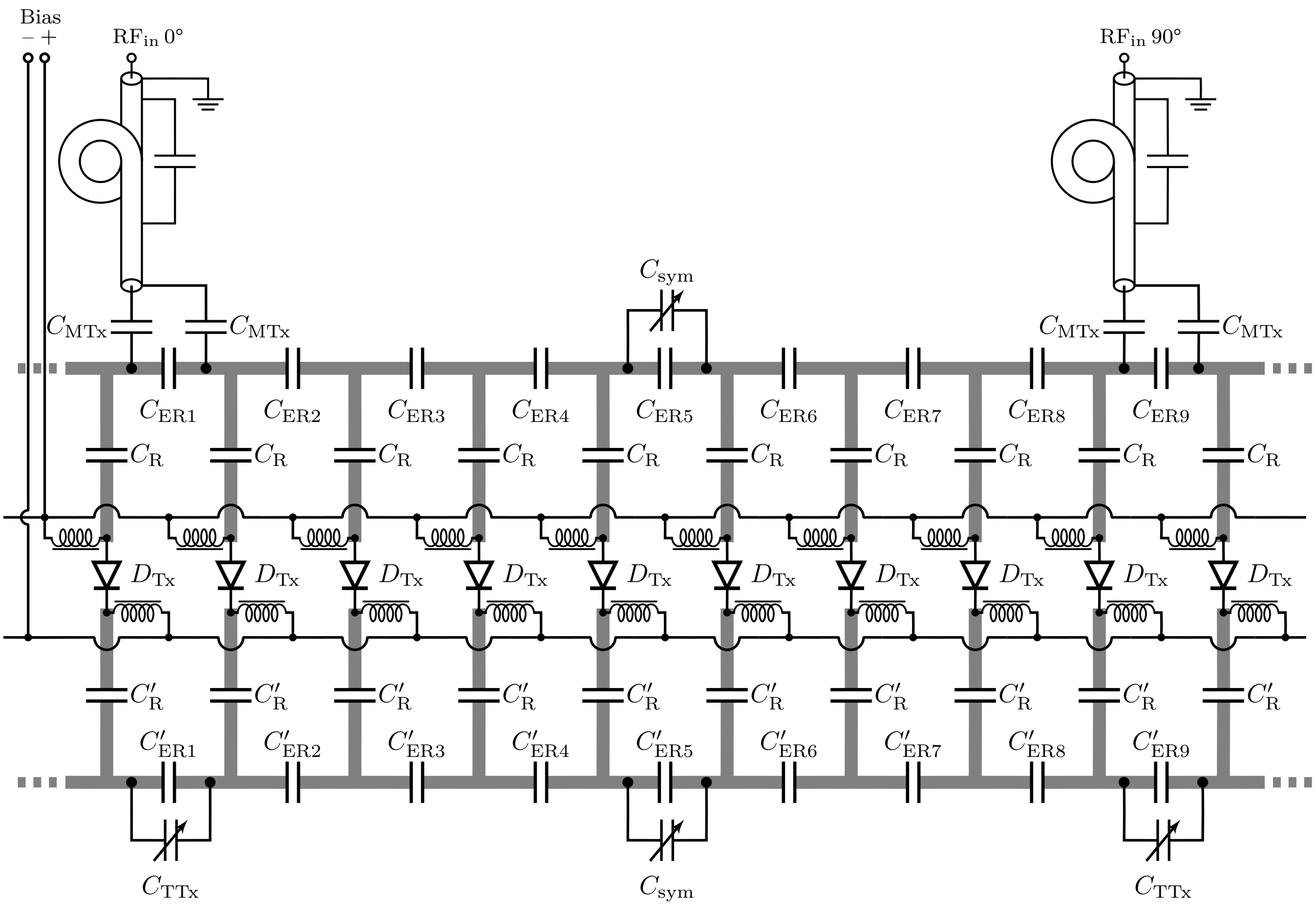

Figure S2: Circuit schematic of the local circularly polarized bandpass Tx birdcage coil. The coil was actively tuned by forwand-biasing the PIN-diodes D_{Tx} via a central ring structure and RF chokes L_{RFC} for each of the 16 rungs. The bias ring was powered with 5 A through a filter panel containing eight toroid coils to prevent interference with the RF fields of the birdcage coil. The tuning of the coil was achieved with the end-ring capacitors C_{ER} and the capacitors at the rungs C_{R} with fine-tuning to the Larmor frequency accomplished using a variable tuning capacitor C_{TTx}. The two transmit ports each contain a solenoid cable trap with parallel capacitors. Matching to 50 Ω was realized using a capacitive matching circuit (variable C_{MTx}) with the agar brain phantom in the Rx coil as a load.

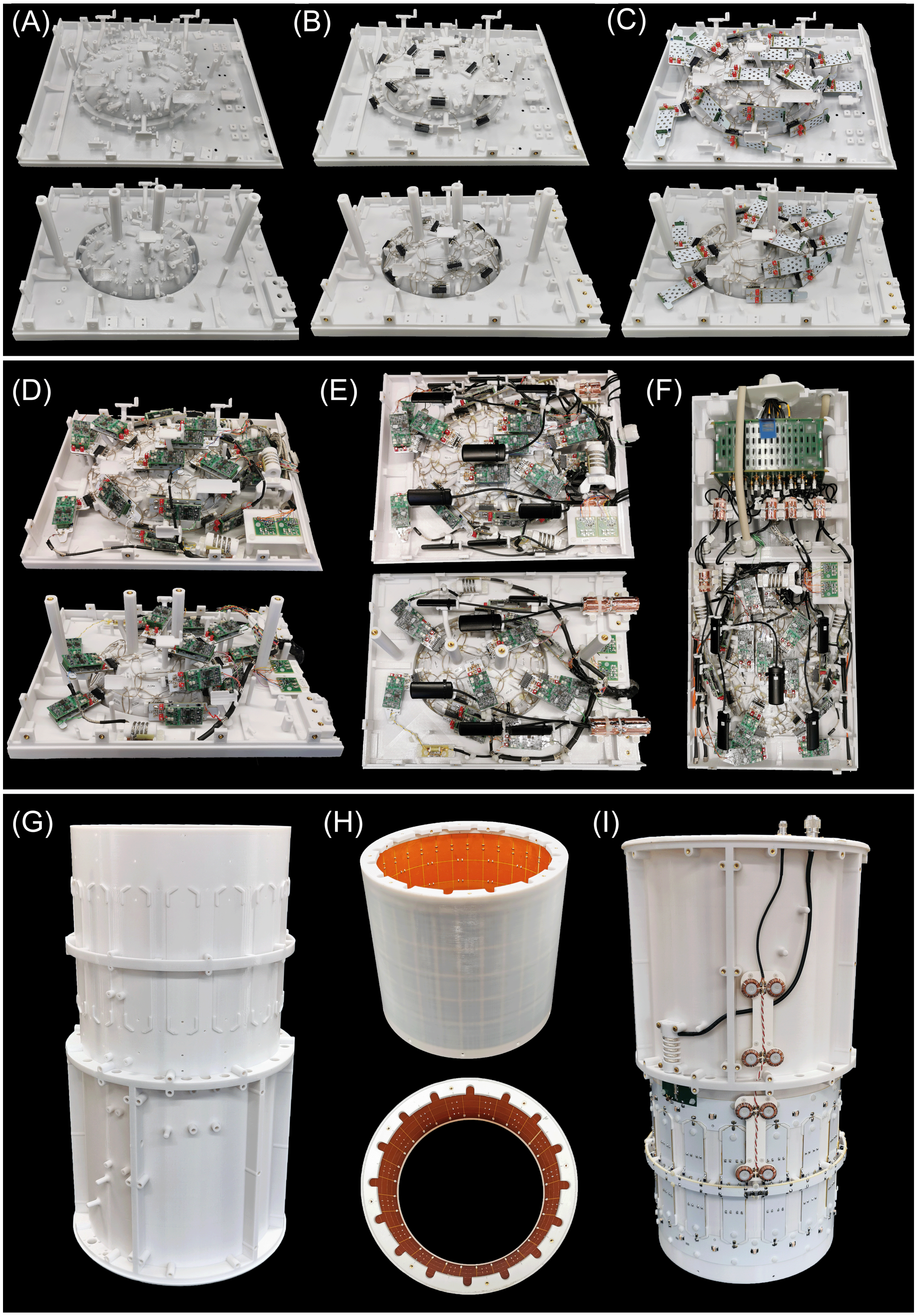

Figure S3: Construction steps for the receive coil (A - F) and transmit coil (G - I). (A) 3D-printed receive coil parts, (B) with loops, (C) with daughter boards, (D) with preamplifiers, wiring and EEPROM chips, (E) with field probes and cable traps and (F) in the assembled arrangement. (G) 3D-printed transmit birdcage coil former. (H) RF-Shield of the birdcage coil comprising multiple copper segments. (I) Assembled circularly polarized birdcage coil consisting of 16 rungs, two endrings, and a central bias ring. Further components are filter panels with toroid RF chokes for bias feeding, solenoid RF cable traps, and matching networks for the two Tx ports.

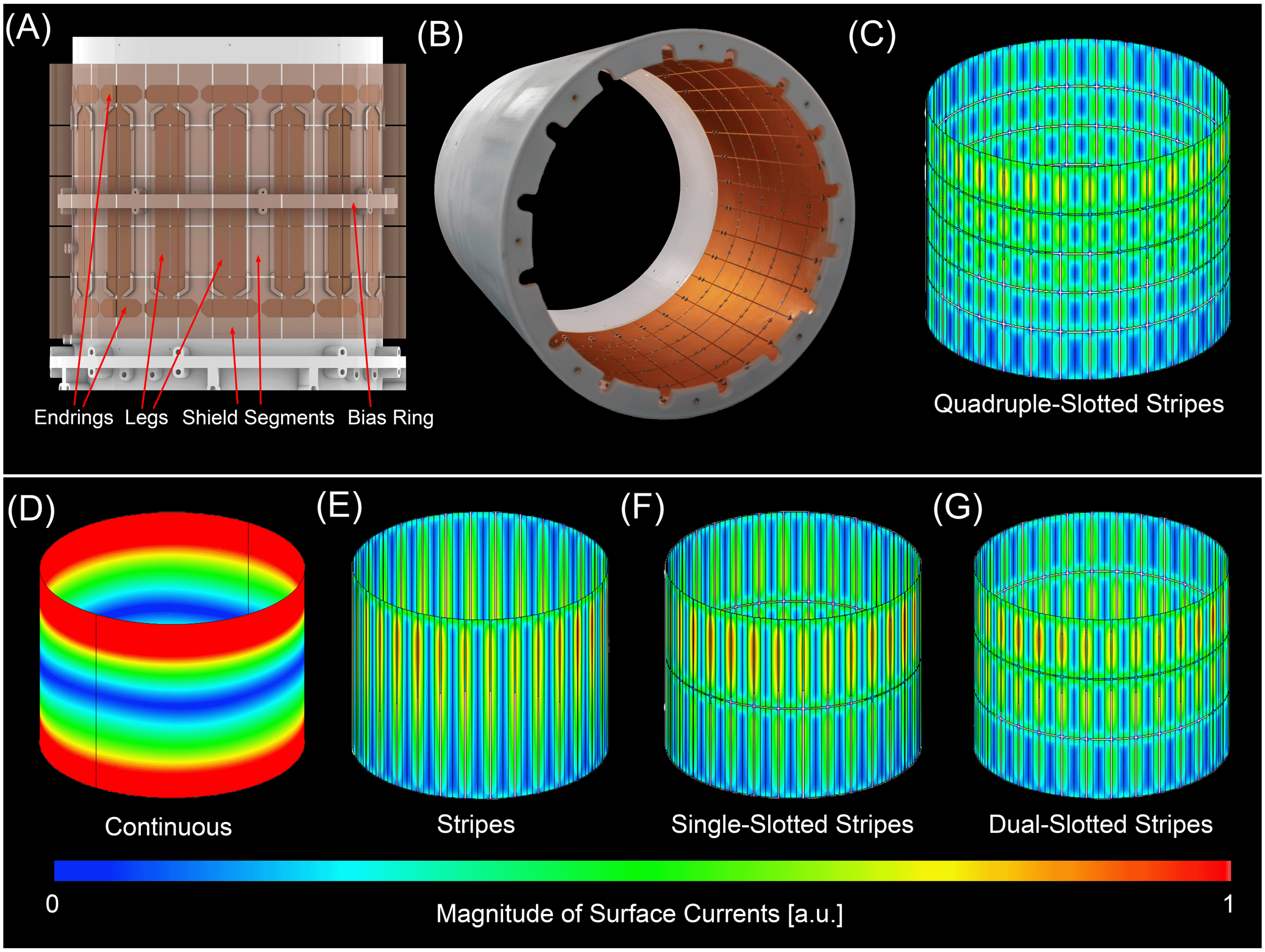

Figure S4: RF shield design for low eddy-current generation using strong gradient systems. (A) Copper structures of the Tx birdcage coil and the RF shield. The constructed RF shield consists of 160 segments made of 9µm-thick copper sheets. Ceramic capacitors (1 nF) were exclusively placed along the mirror current path to provide RF continuity. (B) Close-up photo of the RF shield, laminated to the inner side of the outer tubular coil former. (C) Simulated eddy current distribution of the final shield design (values presented using a relative normalized scale). (D-G) Eddy current simulation for four alternative shield designs that informed the development of the final design.
The final RF shield design C demonstrated a reduction in maximum eddy currents of 76%, 9.1%, 8.6%, and 7.4% compared to the shield layouts D, E, F, and G, respectively.

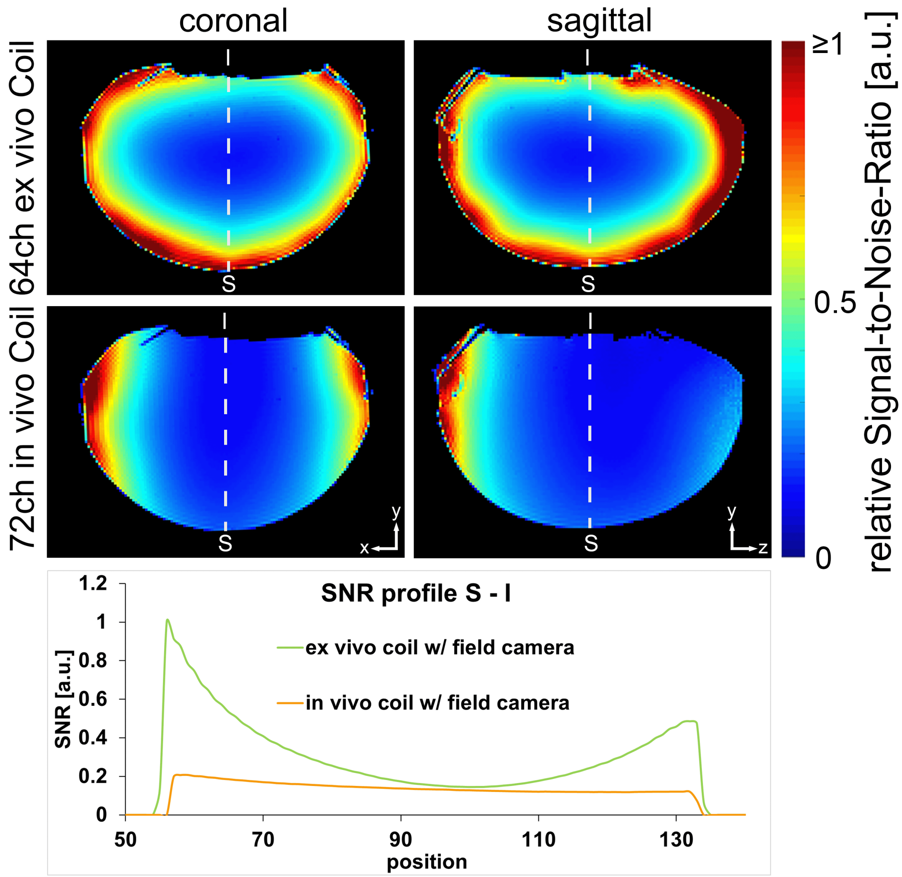

Figure S5: Comparison of flip-angle-corrected signal-to-noise-ratio obtained from the ex vivo coil array and the 72-channel Connectome head coil both equipped with the field camera. Top: SNR maps two representative coronal and sagittal slices. Bottom: SNR profiles extracted along the dashed lines.

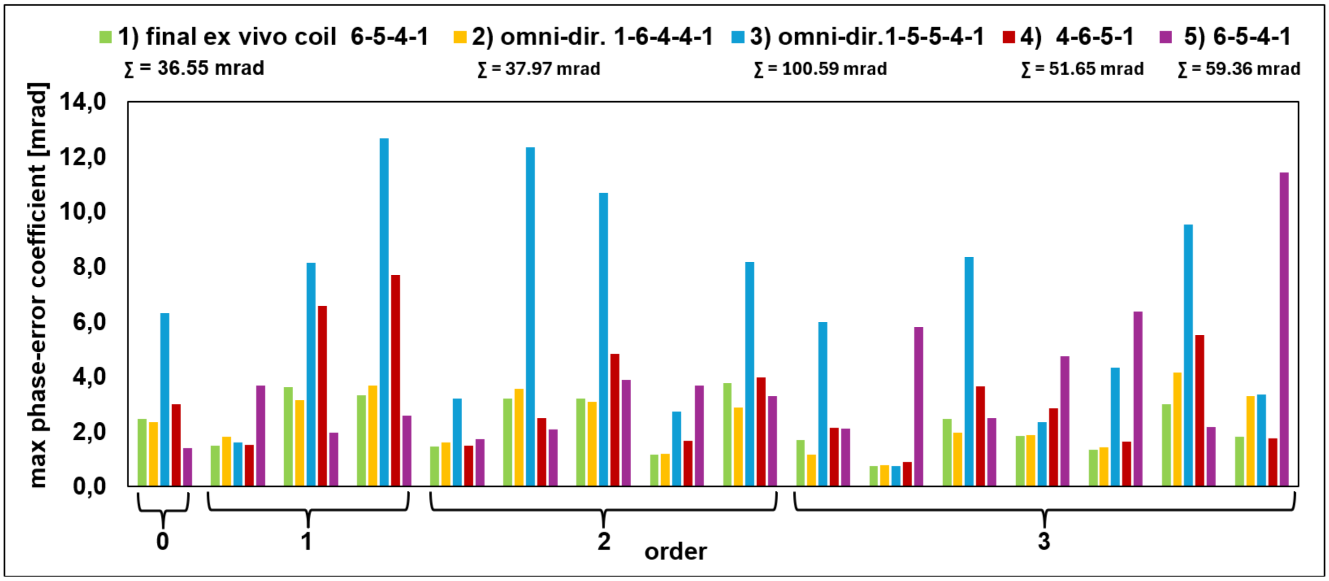

Figure S6: Maximum phase error for various field probe configurations evaluated during the it-erative optimization process. Configuration 1 (green) represents the final implemented probe arrangement, while configurations 2-5 illustrate earlier stages in the optimization process. Con-figurations 3, 4, and 5 show substantial deviations from the final design, resulting in considerably higher maximum phase errors Configuration 2 (yellow), featuring an omnidirectional (omni-dir.) probe distribution that more fully encompasses the brain structure, demonstrates performance comparable to our final implementation but presented significant practical challenges due to cable routing constraints.

The progressive reduction in maximum phase errors across configurations reveals our systematic optimization approach. This process balanced theoretical performance with practical implementation considerations, eventually leading to our final design choice that maintains solid field monitoring capabilities while accommodating the physical constraints of the ex vivo coil assembly.

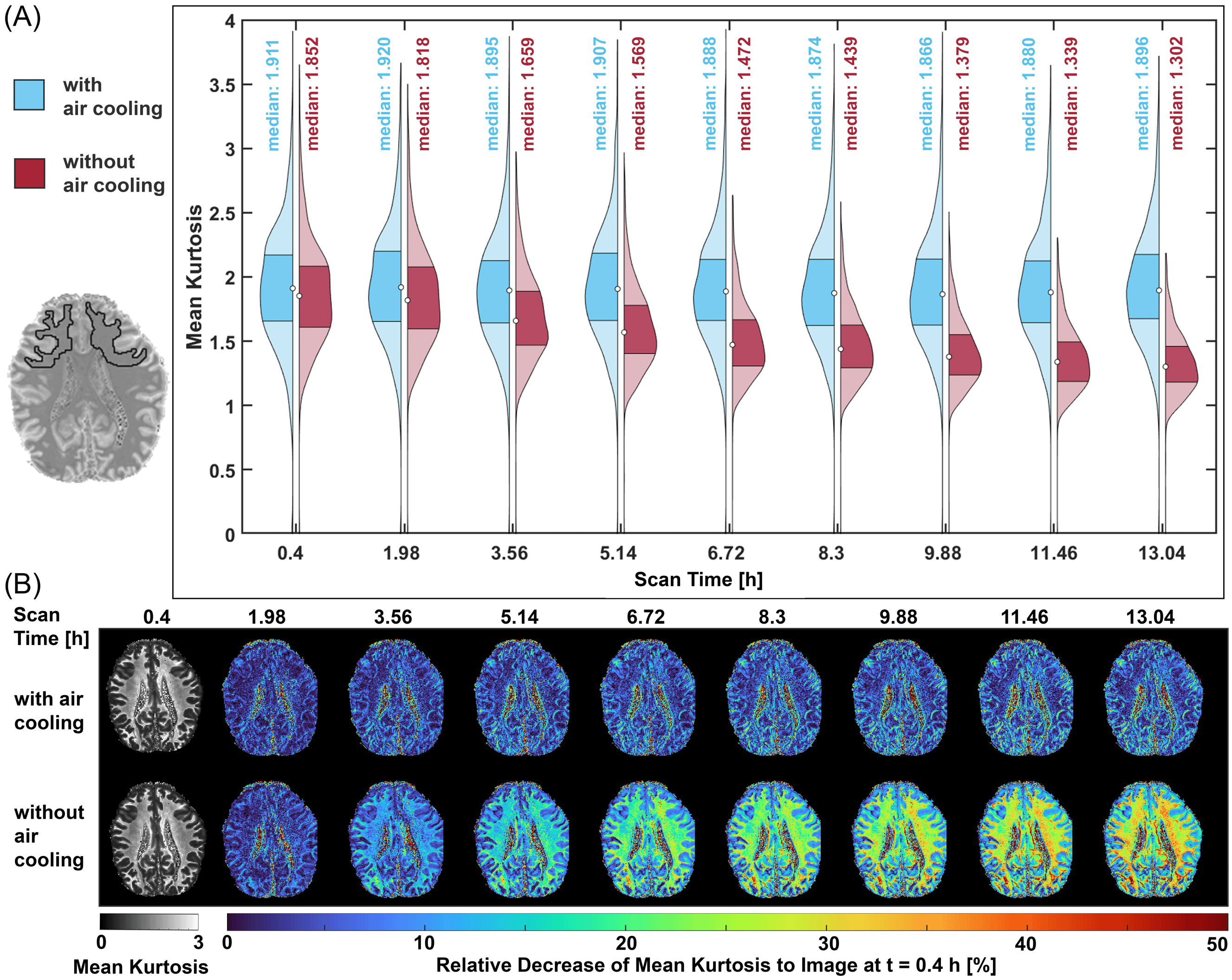

Figure S7: dMRI measurement series with and without forced air cooling, each over 13.04 hours (A) Violin plots of mean kurtosis values at nine time points in ROI of white matter. (B) Change in mean kurtosis relative to a reference image at 0.4 h scan time.
